# Drug Repositioning for Multiresistant *Staphylococcus aureus* and *Escherichia coli* from Pandemic Response Box and Global Health Priority Box of MMV

**DOI:** 10.1021/acsomega.5c12871

**Published:** 2026-08-12

**Authors:** Daniela S. Gonçalves, Nagela B. S. Silva, Anna L. O. Santos, Sara L. de Souza, Gabriel G. Calefi, Ralciane de P. Menezes, Andreia M. de Oliveira, Ghabriel Honório-Silva, Camila M. de Andrade, Marcos P. O. Almeida, Thales A. M. Fernandes, Bellisa de F. Barbosa, Eloísa A. V. Ferro, Robinson Sabino-Silva, Carlos H. G. Martins

**Affiliations:** † Laboratory of Antimicrobial Testing, Institute of Biomedical Sciences, Federal University of Uberlândia, Uberlândia, Minas Gerais 38400-694, Brazil; ‡ Innovation Center in Salivary Diagnostic and Nanobiotechnology, Institute of Biomedical Sciences, Federal University of Uberlândia, Uberlândia, Minas Gerais 38400-694, Brazil; § Laboratory of Immunophysiology of Reproduction, Institute of Biomedical Sciences, Federal University of Uberlândia, Uberlândia, Minas Gerais 38400-694, Brazil; ∥ Laboratory of Applied Toxinology, Butantan Institute, São Paulo 05503-900, Brazil

## Abstract

The phenomenon of antimicrobial resistance (AMR) can occur naturally through spontaneous mutation without major damage; however, the overuse and misuse of antimicrobials is responsible for millions of deaths annually. The development of new drugs is an expensive and slow process, while repositioning active substances to different biological activities is a cheaper and faster option. The aim of this study was to evaluate the possibility of repositioning 640 known chemical compounds provided by the Medicines for Malaria Venture organization for antibacterial and antibiofilm activities against multidrug-resistant *Staphylococcus aureus* (ATCC and clinical isolated) and *Escherichia coli* (ATCC and clinical isolated), their cytotoxicity, and prediction of interaction sites between compounds and bacteria. MMV020752 and MMV1578884 against *S. aureus* ATCC and MMV1579850 against *E. coli* ATCC presented the lowest minimum inhibitory concentration (MIC) of 0.019 μM. MMV1578884 presented the lowest minimum inhibitory concentration of biofilm (MICB_50_) of 0.0095 μM and the lowest inhibitory concentration (IC_50_) of 0.019 μM against *S. aureus* ATCC. MMV1578884, MMV1580854, MMV027339, and MMV1634081 maintain the viability of HFF cells above 100% at 10 and 20 μM, and their interactions with bacteria occur in lipid and protein regions. It is concluded that drugs already available on the market or in more advanced clinical stages can be repositioned for other targets, even other microorganisms.

## Graphical abstract

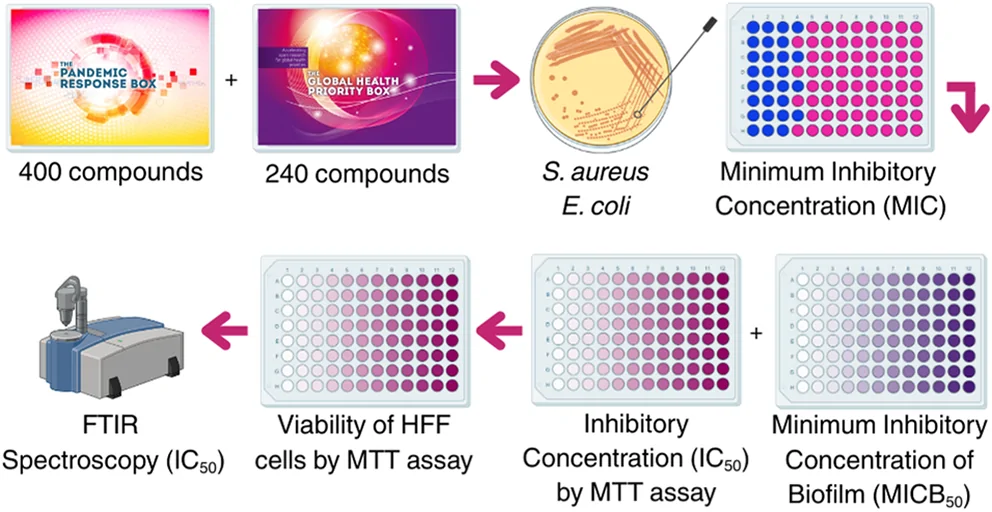

## 1 Introduction

The simplest mechanism of antimicrobial resistance (AMR) is a natural phenomenon resulting from a spontaneous mutation that can appear without contact with any drug, encoded by chromosomes, not transmitted, and with low frequency.^1^ The overuse and misuse of antimicrobials create a selective pressure that can determine the prevalence and transmission of resistance genes, increasing the burden of AMR.^2^

In 2019, 1.27 million deaths were attributable to antibiotic-resistant bacteria, while in 2050 it is estimated that 10 million deaths will be caused by AMR.^3^ In 2020, during the first wave of coronavirus (SARS-CoV-2), more than 70% of patients received an antibiotic even though only 3.5% were diagnosed with community-acquired bacterial infection and 14.3% were diagnosed with hospital-acquired bacterial infection, spreading the AMR global crisis.^4^

According to the World Health Organization Bacterial Priority Pathogens List 2024, carbapenem-resistant *Klebsiella pneumoniae*, third-generation cephalosporin-resistant *Escherichia coli*, and carbapenem-resistant *Acinetobacter baumannii* are at the top of the list of bacteria classified as the critical priority group, and methicillin-resistant *Staphylococcus aureus* (MRSA) is classified as the high-priority group.^5^ Among them, *S. aureus* and *E. coli* are responsible for approximately 50% of the fatal AMR in high-income countries.^6^

*S. aureus* colonizes about 30% of the human population in a commensal interaction with skin and nasal mucosa without harm. However, depending on the disruption of the epithelial barrier and host immune system, it can infect all of the body’s tissues, including bone, lungs, heart, and blood.^7^ MRSA pathogenesis is remarkable because of a diverse array of virulence factors like polysaccharide intercellular adhesin, extracellular DNA, wall teichoic acids, and capsule, which are major components that confer a stable biofilm; an enterotoxin, toxic shock syndrome toxin 1; and exfoliative toxins, leukotoxins, hemolysins, and Protein A.^8^

β-Lactam antibiotics act by binding strongly to penicillin-binding proteins (PBPs), essential proteins in the synthesis of peptidoglycan, inhibiting cell wall synthesis and leading to bacterial lysis.^9^ MRSA possess a transposable genetic element known as staphylococcal chromosomal cassette *mec* (SCC*mec*) that contains *mec*A, *mec*B, *mec*C, or *mec*D genes, which encode a specific PBP2a with low affinity for most β-lactam antibiotics, restoring peptidoglycan synthesis and generating high-level methicillin resistance.^10^

*E. coli* colonizes the human gastrointestinal tract a few hours after birth, in a beneficial interaction without any adverse effects.^11^ However, there are pathovars, including foodborne, of great concern that cause diarrheagenic and extraintestinal diseases such as hemolytic uremic syndrome, bladder infection, lung inflammation, brain membrane inflammation, and sepsis.^12^ Their virulence factors are of the type adhesins, formation and development of biofilm; iron uptake factors to enhance bacterial growth; and protectins responsible for outer membrane proteins and capsular protection or toxins, comprising shiga toxin, cytotoxic necrotizing factor-1, and hemolysins, for example.^13^

Among *E. coli*, resistance to β-lactams is most frequently mediated by β-lactamases, enzymes that hydrolyze the amine bond in the β-lactam ring.^13^ These enzymes can be divided into families of AmpC-β-lactamases, metallo-β-lactamases, OXA-β-lactamases, and extended-spectrum β-lactamases, which are considered the predominant source of *E. coli* resistance against the third and fourth generations of cephalosporins.^14^

The traditional method for discovering new drugs is expensive, time-consuming, and challenging, with an estimated cost of 1.24 billion dollars and a delay of up to 17 years.^15^ Alternatively, finding new biological applications for drugs that already exist on the market, extending to active substances that failed the clinical phase because of their toxicity or insufficient efficacy and drugs withdrawn from the market due to budget, safety concerns, or other reasons, is cheaper and faster, a concept known as drug repositioning.^16^

In this context, the Medicines for Malaria Venture (MMV) is a not-for-profit organization dedicated to preventing and eliminating malaria that, in association with the Drugs for Neglected Diseases initiative, created the Pandemic Response Box (PRB) with 400 compounds^17^ and, in association with the Innovative Vector Control Consortium and Bristol Myers Squibb, created the Global Health Priority Box (GHPB) with 240 compounds.^18^ This joint effort provided scientists worldwide with free access to collections, so-called boxes, composed of antibacterials, antivirals, antifungals, antimalarials, insecticides, and other chemically synthesized compounds already available on the market or in various phases of drug discovery or development, to screen for vector control, transmission-blocking, and new therapies for neglected, zoonotic, and drug-resistant diseases.

Therefore, the objective of this present work was to evaluate the possibility of repositioning PRB and GHPB compounds provided by the MMV organization for antibacterial and antibiofilm activities of multiresistant *S. aureus* and *E. coli*, their cytotoxicity, and prediction of interaction sites between compounds and bacteria.

## 2 Materials and Methods

### 2.1 MMV Compounds

The 640 compounds were received freeze-dried and arranged in 96-well plates. Upon receipt, they were solubilized in 10 μL of dimethyl sulfoxide (DMSO; Sigma-Aldrich, St. Louis, Missouri, USA), as recommended by the MMV organization instructions, resulting in a concentration of 10 mM. The stock solution was then diluted in 90 μL of sterile distilled water to achieve a concentration of 1 mM. Five aliquots of 20 μL were generated and stored at −20 °C for future assays.

### 2.2 Bacterial Strains Used in the Assays

The strains of *S. aureus* subsp. *aureus* ATCC BAA-44, multidrug-resistant, SCC*mec* Type I, and *E. coli* ATCC BAA-198, multidrug-resistant, extended-spectrum β-lactamase (ESBL) TEM-26 were obtained from American Type Culture Collection (ATCC, Manassas, Virginia, USA). The strain of *S. aureus*, multidrug-resistant, clinical isolated (CI) from blood, was provided by Hospital de Clínicas da Faculdade de Medicina de Ribeirão Preto (Ribeirão Preto, São Paulo, Brazil), and *E. coli*, multidrug-resistant, clinical isolated (CI) from a surgical wound, was provided by Hospital de Câncer de Barretos (Barretos, São Paulo, Brazil). All strains were maintained under cryopreservation at −20 °C in Mueller Hinton Broth (MHB) and glycerol 80% (Kasvi, Pinhais, Paraná, Brazil) as part of the culture collection of the Laboratory of Antimicrobial Testing (LEA) of the Federal University of Uberlândia (UFU).

### 2.3 Screening by Minimum Inhibitory Concentration (MIC)

The minimum inhibitory concentration (MIC) is the lowest concentration responsible for limiting bacterial growth and was determined by microdilution broth methodology, as recommended by the Clinical and Laboratory Standards Institute.^19^ This first assay serves as the initial screening of the samples and was performed with all compounds. The aliquot at a concentration of 1 mM was diluted in sterile distilled water to a concentration of 80 μM. Ninety-six-well microplates (Nest, Wuxi, Jiangsu, China) were prepared with MHB, and each compound concentration ranged from 0.0095 to 20 μM. Bacteria cells were standardized on the McFarland 0.5 scale and confirmed in a Densimat densitometer (Biomérieux, Grassina, Florence, Italy) to reach a final concentration of 5 × 10^5^ CFU/mL in well. For each day the experiment was conducted, a microplate was used for quality control analysis, using the same culture medium and inoculum. Vancomycin (Sigma-Aldrich) was used as a positive control for *S. aureus* ranging from 0.08 to 4.07 μM, and tetracycline (Sigma-Aldrich) was used as a positive control for *E. co* *li* ranging from 0.025 to 13.275 μM. Microplates were incubated at 36 °C for 24 h. Sterility controls of the culture medium, DMSO, compounds, and antibiotics alone were performed, in addition to inoculum controls to verify bacterial growth under the experimental conditions.

After incubation, 30 μL of 0.02% aqueous resazurin solution (Sigma-Aldrich) was added to each well to visualize bacterial growth. The interpretation of the results was performed by visual assessment only. The resazurin blue color reveals the absence of bacterial activity, and the resorufin pink color reveals the presence of cellular metabolism.^20^ All assays were performed in triplicate.

### 2.4 Biofilm Formation Inhibition

The inhibition of biofilm formation was evaluated based on the quantification of biomass and cell viability. For this reason, two 96-well microplates were prepared with MHB supplemented with 5% glucose (Nuclear, Diadema, São Paulo, Brazil) under the same MIC conditions. Compounds with promising results in the MIC test were serially diluted in the range from 0.0095 to 20 μM. Vancomycin (Sigma-Aldrich) was used as a positive control for *S. aureus* ranging from 0.08 to 4.07 μM, tetracycline (Sigma-Aldrich) was a positive control for *E. coli* ranging from 0.025 to 13.275 μM, and bacterial cells were prepared to reach 5 × 10^5^ CFU/mL in well. Microplates were incubated at 36 °C for 24 h.

After incubation, one of the plates was used for the quantification of biomass, according to Stepanovic et al.^21^ The contents of the wells were aspirated and carefully washed with sterile distilled water to remove planktonic cells. Next, methanol (Êxodo Científica, Sumaré, São Paulo, Brazil) was added for 20 min to fix the biofilm. The methanol was aspirated, and 2% crystal violet solution (Sigma-Aldrich) was added for 15 min to stain the biofilm. After removing the crystal violet and carefully washing the wells with sterile distilled water, 33% acetic acid (Êxodo Científica) was added for 30 min to resolubilize the biofilm, and absorbance was measured by a Versa Max spectrophotometer (Molecular Devices, San Jose, California, USA) at 490 nm. The minimum inhibitory concentration of biofilm (MICB_50_) is the lowest concentration responsible for limiting 50% or more of biofilm formation, calculated by the equation: 1−(*A*_490_ of the test/*A*_490_ of nontreated control) × 100.^22^

The other plate was used for the quantification of cell viability with [3-(4,5-dimethylthiazol-2-yl)-2,5-diphenyltetrazolium bromide] tetrazolium (MTT; Sigma-Aldrich), according to Grenho et al.^23^ The supernatant of each well was aspirated and carefully washed with sterile distilled water to remove planktonic cells. Next, a 0.5 mg/mL MTT solution was added, and the microplate was incubated again at 36 °C for 2 h. The tetrazolium salt is reduced to insoluble purple formazan by active metabolism of bacteria and, later, solubilized in DMSO for 15 min. The supernatant of each well was aspirated and transferred to a new plate, and absorbance was measured in a Versa Max spectrophotometer (Molecular Devices) at 490 nm. The concentration capable of inhibiting 50% of the viable biofilm cells (IC_50_) was calculated by nonlinear regression using GraphPad Prism version 8.0.2 (Inc., San Diego, California, USA).^24^ All assays were performed in triplicate.

### 2.5 Cytotoxicity in the HFF Culture

Human foreskin fibroblasts (HFF cell line) obtained from ATCC were cultured in 25 or 175 cm^2^ culture flask containing Roswell Park Memorial Institute (RPMI)-1640 medium (Sigma-Aldrich) supplemented with 100 U/mL penicillin (Sigma-Aldrich), 100 μg/mL streptomycin (Sigma-Aldrich), and 5% heat-inactivated fetal bovine serum (FBS; Cultilab, Campinas, São Paulo, Brazil), in a humidified incubator at 37 °C and 5% CO_2_. In accordance with protocol 13/2012 from the Ethics Committee of the Federal University of Uberlândia, the use of commercially acquired cell lines does not require ethical approval.

To evaluate the effect on the viability of HFF cells, compounds with promising results in the MIC test were submitted to the MTT assay, according to Mosmann.^25^ For this purpose, HFF cells in a complete medium were cultured in 96-well microplates (3 × 10^4^ cells/100 μL/well) for 24 h under culture conditions. Next, compounds in the complete medium were added to reach a final concentration of 10 or 20 μM in a well, or 2% DMSO in complete medium was added as a vehicle, or only complete medium was used as a control for untreated cells. Microplates were incubated for 24 h under the same conditions. The supernatants were discarded, and HFF cells were incubated with 0.5 mg/mL MTT in the complete medium for 4 h under culture conditions. MTT was removed, and formazan crystals were dissolved by the addition of 10% sodium dodecyl sulfate (SDS; Sigma-Aldrich) in 50% dimethylformamide (DMF; Sigma-Aldrich). Microplates were incubated at 50 °C for 15 min, followed by agitation. The absorbance was measured in a Versa Max spectrophotometer (Molecular Devices) at 570 nm. The results were expressed as the percentage of viable cells in relation to 2%DMSO considered 100%. All assays were performed in triplicate.

### 2.6 Infrared Spectroscopy: Spectral Data Analysis

Attenuated total reflection Fourier transform infrared spectroscopy (ATR-FTIR) was used to analyze the samples in a portable biophotonic platform (Agilent Cary 630 FTIR, Agilent Technologies, Santa Clara, CA, USA). The ATR system, which incorporates a diamond internal reflection element (IRE), enables the efficient acquisition of infrared spectra within the 4000–650 cm^–1^ range, providing detailed molecular information on functional groups based on their characteristic vibrational modes. As a first step, the infrared spectrum of each bacterial strain was recorded individually. For this, bacterial cultures were diluted in ultrapure type I water (MiliQ) to neutralize potential interference from other components at a concentration of 2.7 × 10^9^ CFU/mL. Three microliters of the bacterial dilution was directly applied to the crystal and allowed to dehydrate under airflow for 10 min before ATR-FTIR spectrum acquisition. Then, 3 μL of each compound with promising results in the MIC test at a concentration of 1 mM followed the same procedure individually. To assess interactions between bacteria and MMV compounds, both were incubated together for 24 h, maintaining the same volume and conditions as previous spectral acquisition. Infrared spectra were recorded at a resolution of 2 cm^–1^ with 64 scans. Second-derivative spectra were generated based on the raw data using Origin Pro 9.0 (OriginLab, Northampton, MA, USA), employing the Savitzky–Golay algorithm with a second-order polynomial and 20-point window for smoothing.^26^

### 2.7 *In Silico* Analysis

In order to shed light on the potential antibacterial mechanism, we performed molecular docking simulations of the most promising results. The target structures from *S. aureus* were collected from AlphaFold-DB retrieved from UniProt.^27,28^ In addition, the targets from *E. coli* were defined according to Mondal et al., which identified potential targets by extensive computational analysis.^29^ In parallel, the structures of the compounds were obtained from PubChem.^30^ The molecular docking simulations were performed using Dockthor v.2 with a grid box of 20 Å for atom selection from the annotated binding sites present in UniProt. The docking was ranked by the total energy calculated from the MMFF94S force field.^31^ The results were visualized using matplotlib, pandas, and seaborn Python (https://www.python.org/) packages.^32–34^ The interactions were analyzed using Discovery Studio Visualizer v. 24.1.0 (BIOVIA-Dassault Systèmes, San Diego, California, USA).

**Table 1:** Minimum Inhibitory Concentrations (MICs) in μM of PRB and GHPB Compounds against *S. aureus* and *E. coli*

compound ID	PubChem ID	*S. aureus* ATCC BAA-44	*S. aureus* clinical isolated	*E. coli* ATCC BAA-198	*E. coli* clinical isolated
Pandemic Response Box					
MMV1580173	5583	5	2.5	-	-
MMV1580853	413628	0.31	1.25	-	-
MMV020752	2392	0.019	5	-	-
MMV1578575	3000226	0.31	1.25	-	-
MMV1593541	71456558	1.25	0.62	-	-
MMV1593537	11385393	5	1.25	-	-
MMV1634391	23730143	2.5	5	-	-
MMV688756	465951	1.25	2.5	-	-
MMV1580849	14570151	2.5	1.25	-	-
MMV1578884	16744283	0.019	0.078	-	-
MMV1782403	45484770	1.25	1.25	-	-
MMV1580854	59191218	-	-	0.62	0.62
MMV1578566	46836890	-	-	2.5	1.25
MMV1633674	6918462	-	-	1.25	2.5
MMV002612	5426	-	-	1.25	5
MMV1579850	461399	-	-	0.019	5
Global Health Priority Box					
MMV027339	67788	2.5	2.5	-	-
MMV1634081	3017599	2.5	2.5	-	-
MMV1795508	^a^	5	5	-	-
MMV1828776	^a^	2.5	2.5	-	-
Positive Control					
Vancomycin	14969	1.017	0.50	^b^	^b^
Tetracycline	54675776	^b^	^b^	1.66	3.32

a No PubChem ID. -: indicates MIC value >5 μM, which is considered inactive.

b Antibiotics not used.

## 3 Results

With the aim of screening the antibacterial activity of the 640 compounds from PRB and GHPB libraries against *S. aureus* (ATCC and CI) and *E. coli* (ATCC and CI), the minimum inhibitory concentration (MIC) assay was performed first. Twenty compounds selected as hits (15 against *S. aureus* and 5 against *E. coli*) were then tested for their ability to inhibit biofilm formation, since biofilm is one of the mechanisms of bacterial defense and virulence. These hits were also tested for their cytotoxicity in HFF culture as a first step toward assessing the safety of these compounds when they are in contact with human cells. Two hits from the PRB box and two hits from the GHPB box with cellular viability above 100% were investigated regarding their site of interaction with bacteria and mechanisms of action through infrared spectroscopy: spectral data analysis and *in silico* analysis, respectively.

### 3.1 Minimum Inhibitory Concentration (MIC)

Due to the large number of compounds available in PRB and GHPB libraries, all results obtained in this initial screening are presented in Table S1 (Supporting Information). MIC values varied from ≤0.0095 to >20 μM. From PRB, MMV020752 and MMV1578884 against *S. aureus* ATCC and MMV1579850 against *E. coli* ATCC presented the lowest determined concentration of 0.019 μM. Vancomycin and tetracycline, as positive controls, varied from 0.5 to 3.30 μM. Compounds that presented MIC ≤5 μM for *S. aureus* (ATCC and CI) or *E. coli* (ATCC and CI) were considered promising in this first test and selected to proceed to the next tests. Table 1 demonstrates the promising results.

### 3.2 Biofilm Formation Inhibition

In relation to the quantification of biomass, MICB_50_ values varied from 0.0095 to >20 μM, while in relation to the quantification of cell viability, IC_50_ values varied from 0.019 to >20 μM. MMV1578884 (PRB) presented the lowest value for MICB_50_ at 0.0095 μM in accordance with the lowest value for IC_50_ at 0.019 μM against *S. aureus* ATCC. For *E. coli* CI, MMV1580854 (PRB) presented the lowest MICB_50_ of 2.5 μM in accordance with the lowest IC_50_ of 2.3 μM. *E. coli* ATCC was unable to form a biofilm on the microplate. MMV1634081 (GHPB) presented the lowest values of MICB_50_ (1.25 and 2.5 μM) and IC_50_ (0.83 and 1.68 μM) against *S. aureus* ATCC and CI, respectively. Table 2 demonstrates all of the results.

**Table 2:** Minimum Inhibitory Concentration of Biofilm (MICB_50_) and Inhibitory Concentration (IC_50_) in μM of PRB and GHPB Compounds

		*S. aureus* ATCC BAA-44		*S. aureus* clinical isolated		*E. coli* clinical isolated	
compound ID	trivial name	MICB_50_	IC_50_	MICB_50_	IC_50_	MICB_50_	IC_50_
Pandemic Response Box							
MMV1580173	Trimetrexate	20	9.07	20	>20	-	-
MMV1580853	^a^	20	5.59	20	13.07	-	-
MMV020752	Pibenzimol	20	4.82	20	>20	-	-
MMV1578575	Fusidic acid	0.019	0.03	0.0095	1.08	-	-
MMV1593541	^a^	20	14.72	20	>20	-	-
MMV1593537	^a^	5	3.49	0.0095	0.02	-	-
MMV1634391	MGB-BP-3	>20	>20	20	>20	-	-
MMV688756	Sutezolid	10	8.25	5	4.50	-	-
MMV1580849	^a^	10	5.99	20	>20	-	-
MMV1578884	CRS-3123	0.0095	0.019	0.0095	0.58	-	-
MMV1782403	^a^	20	>20	20	3.14	-	-
MMV1580854	^a^	-	-	-	-	2.50	2.30
MMV1578566	Epetraborole	-	-	-	-	10	10
MMV1633674	Retapamulin	-	-	-	-	20	>20
MMV002612	Thalidomide	-	-	-	-	>20	>20
MMV1579850	Sitafloxacin	-	-	-	-	10	11.34
Global Health Priority Box							
MMV027339	Flucofuron	1.25	1.53	2.50	5.97	-	-
MMV1634081	Fenoxacrim	1.25	0.83	2.50	1.68	-	-
MMV1795508	^a^	20	11.76	20	12.69	-	-
MMV1828776	^a^	10	6.03	10	6.13	-	-
Positive Control							
Vancomycin		1.017	0.62	0.50	0.60	^b^	^b^
Tetracycline		^b^	^b^	^b^	^b^	3.32	2.16

a No trivial name. -: MIC value >5 μM considered inactive.

b Antibiotics not used.

### 3.3 Cytotoxicity in the HFF Culture

Among the tested compounds, MMV1578575, MMV1634391, MMV688756, MMV1580849, MMV1578884, MMV1782403, MMV1580854, MMV1578566, MMV002612, and MMV1579850 from PRB and MMV027339 and MMV1634081 from GHPB maintained the cellular viability above 100% at 10 and 20 μM. Table 3 demonstrates all of the results.

**Table 3:** Viability of HFF Cells under PRB and GHPB Compounds

	percentage of viable cells	
compound ID	10 μM	20 μM
Pandemic Response Box		
MMV1580173	78.35 ± 0.32 (*n* = 4)	78.79 ± 1.29 (*n* = 4)
MMV1580853	106.55 ± 3.74 (*n* = 3)	93.52 ± 3.20 (*n* = 3)
MMV020752	95.63 ± 2.38 (*n* = 3)	95.36 ± 0.16 (*n* = 3)
MMV1578575	109.85 ± 1.14 (*n* = 3)	101.53 ± 3.90 (*n* = 3)
MMV1593541	77.93 ± 1.63 (*n* = 3)	32.87 ± 5.20 (*n* = 3)
MMV1593537	123.91 ± 1.51 (*n* = 5)	6.13
MMV1634391	121.23 ± 0.73 (*n* = 5)	112.49 ± 2.32 (*n* = 5)
MMV688756	107.28 ± 1.41 (*n* = 3)	100.27 ± 5.91 (*n* = 3)
MMV1580849	120.85 ± 1.75 (*n* = 5)	112.70 ± 3.30 (*n* = 5)
MMV1578884	108.73 ± 1.94 (*n* = 5)	102.37 ± 4.49 (*n* = 3)
MMV1782403	119.70 ± 4.37 (*n* = 5)	109.50 ± 3.00 (*n* = 5)
MMV1580854	103.43 ± 3.18 (*n* = 4)	112.53 ± 4.14 (*n* = 4)
MMV1578566	107.99 ± 5.67 (*n* = 4)	108.89 ± 2.02 (*n* = 4)
MMV1633674	72.11 ± 3.58 (*n* = 3)	101.61 ± 11.60 (*n* = 3)
MMV002612	102.85 ± 4.74 (*n* = 4)	100.23 ± 0.75 (*n* = 4)
MMV1579850	112.40 ± 2.02 (*n* = 5)	107.24 ± 1.99 (*n* = 5)
Global Health Priority Box		
MMV027339	120.26 ± 3.28 (*n* = 5)	102.27 ± 12.72 (*n* = 3)
MMV1634081	116.74 ± 2.66 (*n* = 5)	109.12 ± 2.13 (*n* = 5)
MMV1795508	82.30 ± 5.75 (*n* = 4)	3.98 ± 0.17 (*n* = 4)
MMV1828776	47.41 ± 7.36 (*n* = 4)	5.06 ± 0.44 (*n* = 4)
Control		
Vehicle (2% DMSO)	100	

### 3.4 Infrared Spectroscopy: Spectral Data Analysis

Infrared spectra were acquired for the 20 hits and their respective bacteria, and a general overview of the results is provided in Figures S1–S4 (Supporting Information). Since, from PRB, MMV1578884 and MMV1580854 presented the lowest concentrations against *S. aureus* or *E. coli*, from GHPB, MMV027339 and MMV1634081 presented the lowest concentrations against *S* *. aureus*, and all of them presented cellular viability above 100%, they were selected for further analysis.

The analysis of full-range ATR-FTIR spectra, complemented by second-derivative transformation, enabled the detection of subtle vibrational mode shifts and offered insight into potential molecular interactions and biochemical alterations induced by the treatments. Tentative assignments of the observed vibrational bands, along with their respective biochemical interpretations, are summarized in Table 4 based on the reference literature and established spectroscopic criteria for biological systems.^35^

**Table 4:** Band Assignment Correspondence of the Spectral Values Indicating the Biomolecules to which Each Wavenumber Corresponds Found for Each Second-Derivative Spectrum Related to the Data Plotted in Figures 1 and 2

wavenumber	tentative band assignment	biomolecular components
1237 cm^–1^	Amide III	Proteins
1238 cm^–1^	Amide III	Proteins
1513 cm^–1^	Amide II	Proteins
1515 cm^–1^	Amide II	Proteins
1638 cm^–1^	Amide I	Proteins
1639 cm^–1^	Amide I	Proteins
1655 cm^–1^	Amide I	Proteins
1783 cm^–1^	Aldehydes, ketones and hydroperoxides	Lipids
1794 cm^–1^	Aldehydes, ketones and hydroperoxides	Lipids
2810 cm^–1^	Stretching vibrations of CH_2_ and CH_3_	Lipids
2817 cm^–1^	Stretching vibrations of CH_2_ and CH_3_	Lipids
2872 cm^–1^	C–H stretching	Lipids
2925 cm^–1^	C–H stretching	Lipids
2932 cm^–1^	C–H stretching	Lipids
2958 cm^–1^	C–H stretching	Lipids
2960 cm^–1^	C–H stretching	Lipids

From PRB (Figure 1), the full spectrum of *S. aureus* treated with MMV1578884 (Figure 1A) demonstrated molecular-level interactions with the ATCC strain, whereas the second-derivative spectra (Figure 1B,C) revealed pronounced spectral variations. Similar patterns were observed for the clinical strain (Figure 1D–F). Vibrational modes at 1794 and 1783 cm^–1^ are found within a spectral region tentatively associated with carbonyl-containing compounds, such as aldehydes, ketones, and hydroperoxides, functional groups commonly reported as products of lipid peroxidation. The observed variations in this region may indicate molecular alterations in lipid structures, potentially reflecting interactions between the tested compound and the bacterial membrane components. Furthermore, the shifts at 2817 and 2810 cm^–1^, which are tentatively assigned to CH_2_ and CH_3_ stretching vibrations of lipid-associated moieties, may reflect subtle modifications in the organization or environment of membrane lipids, potentially affecting lipid–protein interactions within the bacterial membrane.

**Figure 1:**
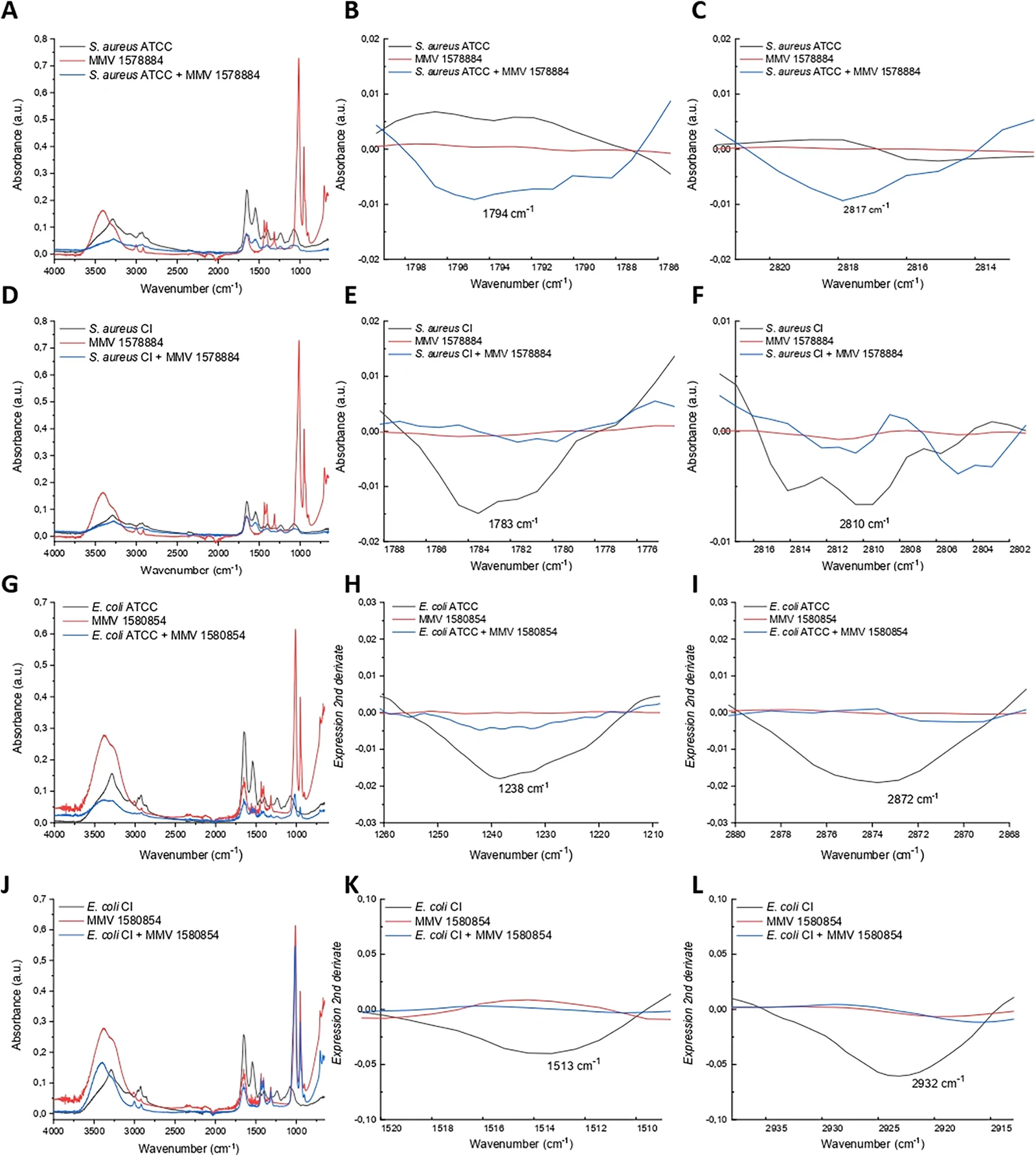
ATR-FTIR analysis reveals molecular shifts in the conformations of *S. aureus* and *E. coli* alone and upon exposure to MMV compounds of PRB. Representative infrared spectra are displayed for bacterial samples (black line), MMV compounds (red line), and bacterial samples incubated with MMV compounds (blue line). (A) Full raw infrared spectrum for *S. aureus* ATCC, MMV1578884, and the bacterial-MMV complex. (B, C) The corresponding second-derivative spectra highlighting spectral shifts. (D) Full raw infrared spectrum for *S. aureus* CI, MMV1578884, and the bacterial-MMV complex. (E, F) The corresponding second-derivative spectra highlighting spectral shifts. (G) Full raw infrared spectrum for *E. co* *li* ATCC, MMV1580854, and the bacterial-MMV complex. (H, I) The corresponding second-derivative spectra highlighting spectral shifts. (J) Full raw infrared spectrum for *E. coli* CI, MMV1580854, and the bacterial-MMV complex. (K, L) The corresponding second-derivative spectra highlighting spectral shifts. Second-derivative spectra were derived from the raw data and processed using the Savitzky–Golay algorithm with a polynomial order of 2 and a 20-point window.

For *E. coli* ATCC and CI treated with MMV1580854, the full spectrum (Figure 1G,J, respectively) showed molecular-level interactions, while the second-derivative spectra (Figure 1H,I,K,L, respectively) exposed distinct spectral variations. Vibrational modes at 1238 and 1513 cm^–1^, assigned to amide III and amide II protein vibrations, respectively, suggest interactions with bacterial structural proteins. Moreover, the shifts at 2872 and 2932 cm^–1^, associated with C–H stretching in lipid moieties, imply potential modifications in lipid–protein interactions within the bacterial membrane.

From GHPB (Figure 2), the treatment of *S. aureus* with MMV027339 revealed molecular-level interactions in the full spectrum of the ATCC strain (Figure 2A), with the second-derivative spectra (Figure 2B,C) highlighting distinct variations. Comparable features were detected in the clinical strain (Figure 2D–F). Vibrational modes at 1237 correspond to the amide III region and at 1515 cm^–1^ to amide II, while peaks at 1655 and 1638 cm^–1^ represent amide I, collectively indicating alterations in bacterial protein structures during treatment. In addition, the peak at 2960 cm^–1^ corresponds to C–H stretching in the lipid region, suggesting modifications in lipid–protein interactions within the bacterial membrane.

**Figure 2:**
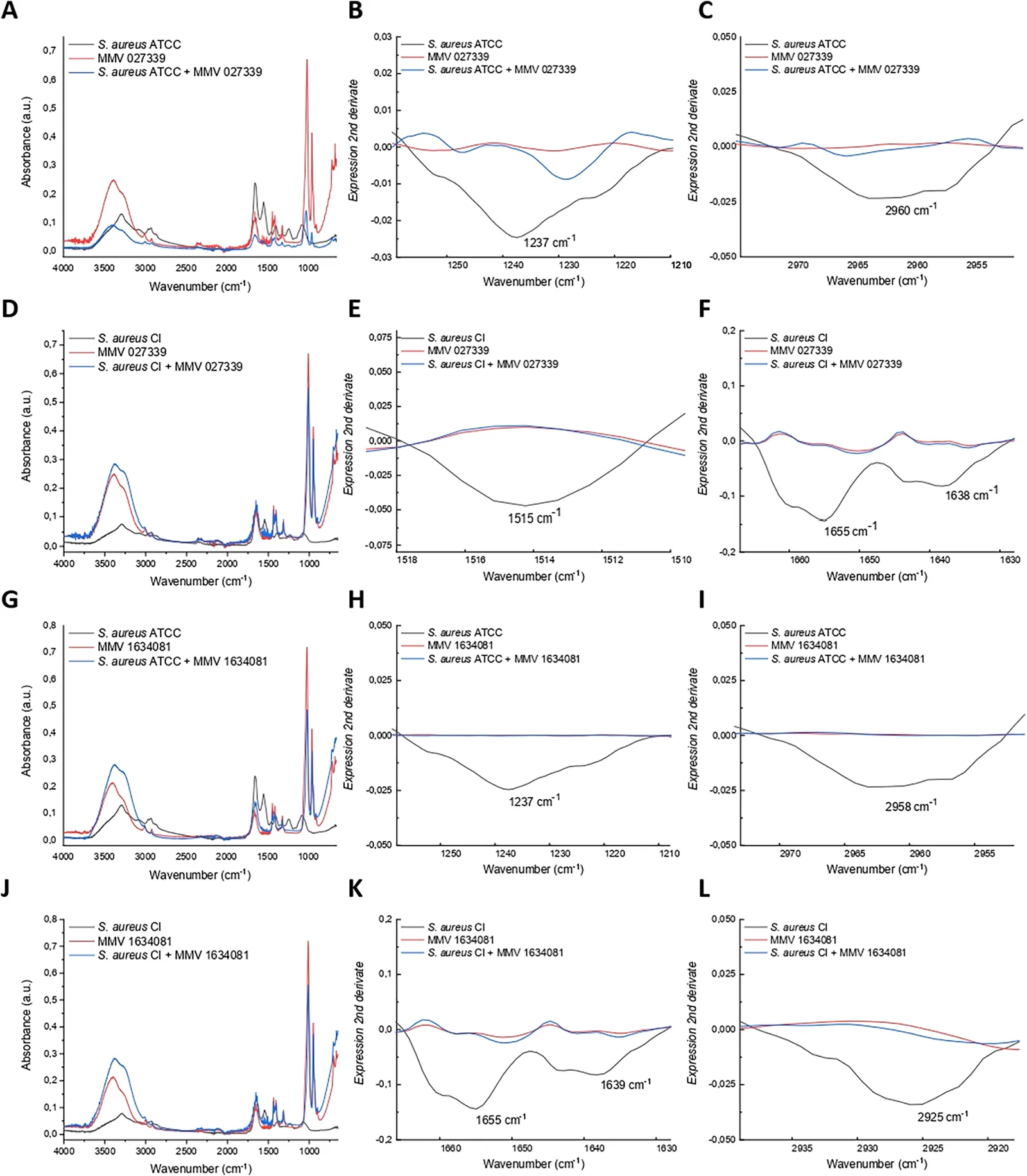
ATR-FTIR analysis reveals molecular shifts in the conformations of *S. aureus* alone and upon exposure to MMV compounds of GHPB. Representative infrared spectra are displayed for bacterial samples (black line), MMV compounds (red line) and bacterial samples incubated with MMV compounds (blue line). (A) Full raw infrared spectrum for *S. aureus* ATCC, MMV027339, and the bacterial-MMV complex. (B, C) The corresponding second-derivative spectra highlighting spectral shifts. (D) Full raw infrared spectrum for *S. aureus* CI, MMV027339, and the bacterial-MMV complex. (E, F) The corresponding second-derivative spectra highlighting spectral shifts. (G) Full raw infrared spectrum for *S. aureus* ATCC, MMV1634081, and the bacterial-MMV complex. (H, I) The corresponding second-derivative spectra highlighting spectral shifts. (J) Full raw infrared spectrum for *S. aureus* CI, MMV1634081, and the bacterial-MMV complex. (K, L) The corresponding second-derivative spectra highlighting spectral shifts. Second-derivative spectra were derived from the raw data and processed using the Savitzky–Golay algorithm with a polynomial order of 2 and a 20-point window.

Finally, for *S. aureus* treated with MMV1634081, the full spectrum (Figure 2G) revealed molecular-level interactions in the ATCC strain, with the second-derivative spectra (Figure 2H,I) revealing distinct spectral variations. Similar features were observed in the clinical strain (Figure 2J–L). The recurrence of peaks at 1655, 1639, and 1237 cm^–1^ indicates structural alterations in bacterial proteins, while the peaks at 2925 and 2958 cm^–1^ confirm changes in the lipidic components of the bacterial membrane.

### 3.5 *In Silico* Analysis

To gain insights into the antibacterial mechanism, we evaluated the binding affinity for *S. aureus* and *E. coli* targets through molecular docking simulations as demonstrated in Figures 3 and 4. Our results showed that MV1634081 and MMV027339 exhibited the best binding affinity for *S. aureus* metalloproteinase aureolysin (aur), while MMV1578884 presented the best binding affinity for *S. aureus* nitric oxide synthase oxygenase (nos) (Figure 3A,B), forming hydrophobic, electrostatic, and hydrogen-bonding interactions with amino acid residues involved in substrate binding and catalysis (Figure 4A–F). In parallel, MMV1580854 exhibited the best binding affinity for *E. coli* aspartate-ammonia ligase (asnA) (Figure 3C,D), forming hydrophobic, electrostatic, and hydrogen-bonding interactions with amino acid residues involved in catalysis (Figure 4G,H).

**Figure 3:**
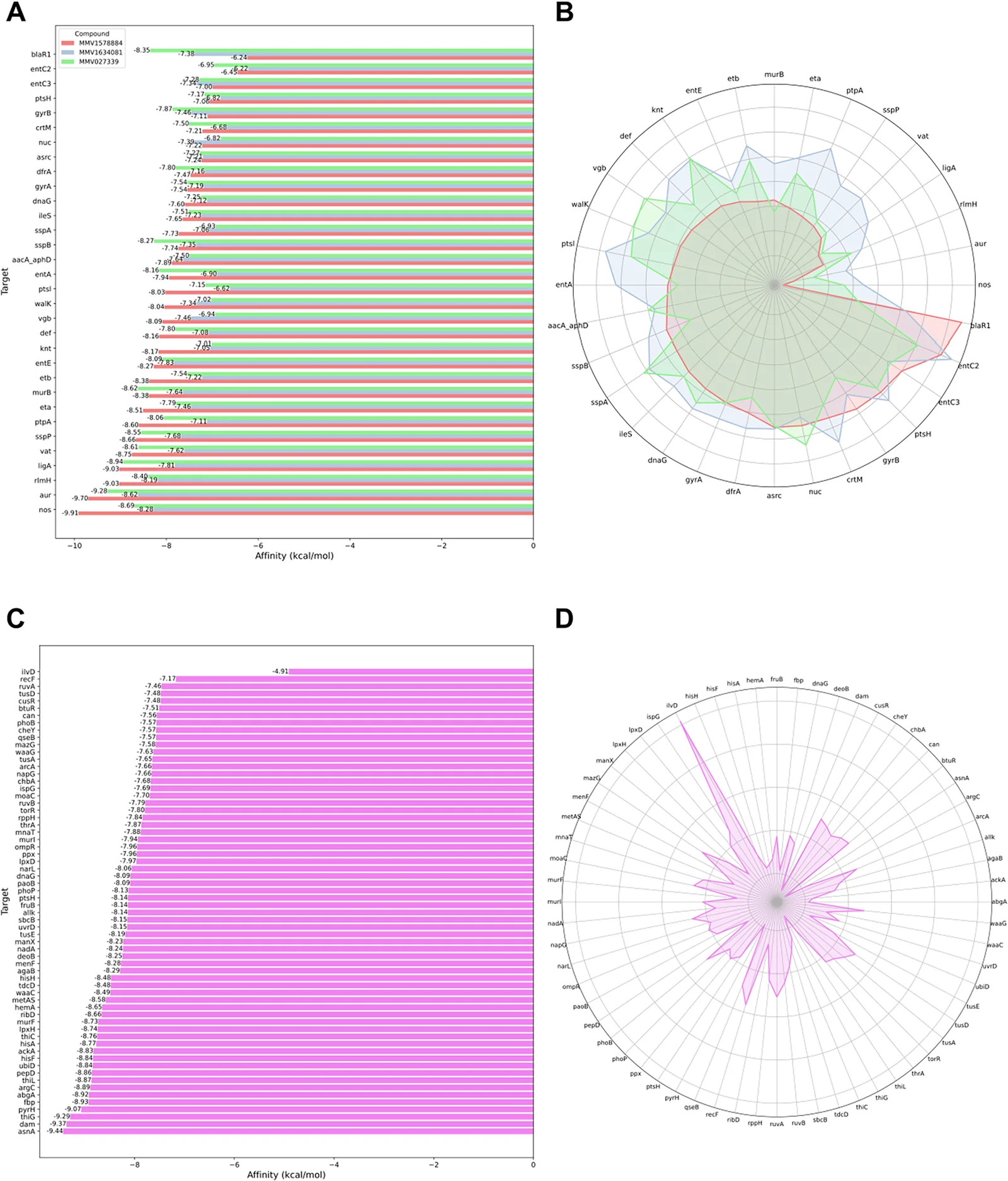
Binding affinity MMV1578884, MMV1634081, and MMV027339 for *S. aureus* and MMV1580854 for *E. coli*. (A, B) Bar plot and radar plot, respectively, of the binding affinity (kcal/mol) of the ligands for *S. aureus* targets. (C, D) Bar plot and radar plot, respectively, of the binding affinity (kcal/mol) of MMV1580854 for *E. coli* targets.

**Figure 4:**
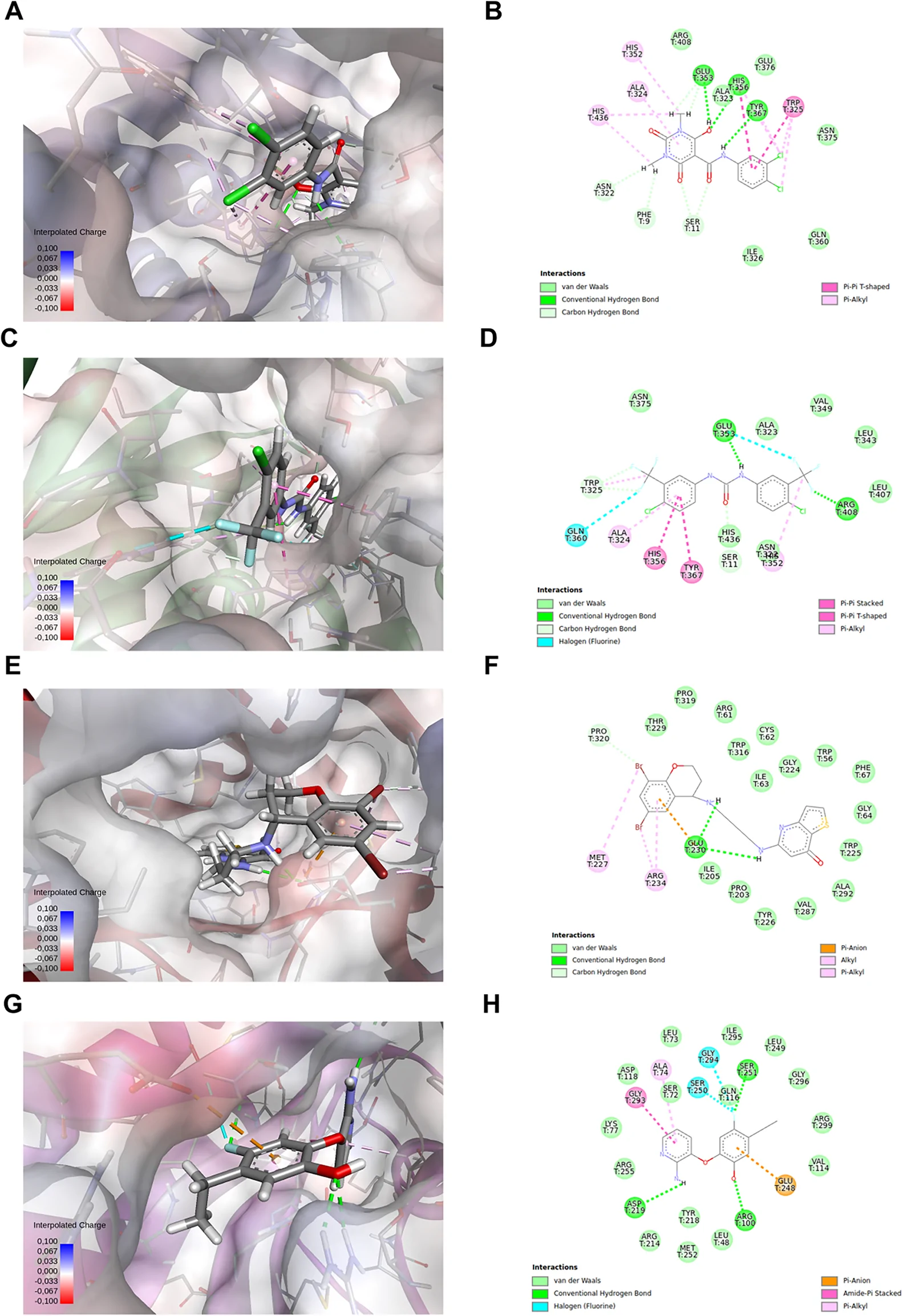
Interactions between MMV1634081, MMV027339, and MMV1578884 for *S. aureus* and MMV1580854 for *E. coli*. (A, B) Binding of MMV1634081 to *S. aureus* metalloproteinase aureolysin (aur). (C, D) Binding of MMV027339 to *S. aureus* metalloproteinase aureolysin (aur). (E, F) Binding of MMV1578884 to *S. aureus* nitric oxide synthase oxygenase (nos). (G, H) Binding of MMV1580854 to *E. coli* aspartate-ammonia ligase (asnA).

## 4 Discussion

Similar results to MIC values with MMV compounds were found in the literature. From GHPB, MMV1795508 (antimalarial) exhibited potent action against *S. aureus* ATCC 29213 at 10 μM.^36^ From PRB, MMV1580853 at 10 μM, MMV1578575 (fusidic acid) at 5 μM, and MMV020752 (pibenzimol) at 1 μM presented activity against *Pseudomonas aeruginosa* PAO1.^37^ MMV1580854 and MMV1578566 (epetraborole) inhibited pan-drug-resistant *Klebsiella pneumoniae* at ≥2 μM.^38^ MMV1580173 (trimetrexate), MMV1580854, MMV1593541, MMV1579850 (sitafloxacin), and epetraborole inhibited the growth of *Acinetobacter baumannii* ATCC 19606 and BC-5 at ≤25 μM.^39^

Of the aforementioned compounds, fusidic acid is already commercially known for Gram-positive bacteria that inhibits the elongation factor G, essential for protein synthesis.^40^ Pibenzimol (Hoechst 33258 or bisbenzimidazole) is commonly used as a fluorescent dye that binds to DNA and inhibits replication,^41^ while trimetrexate inhibits the enzyme dihydrofolate reductase, mainly used for the treatment of *Pneumocystis jirovecii*.^42^ They demonstrated activity against *S. aureus* in the present study, differently from their original bioactivities. Epetraborole is a clinical candidate for Gram-negative infections and inhibits the *Mycobacterium abscessus* leucyl-tRNA synthetase;^43^ sitafloxacin is a broad-spectrum fluoroquinolone used as a third-line antibiotic against *Helicobacter pylori* and second- or third-line treatment against urethritis caused by both *Mycoplasma genitalium* and *Chlamydia trachomatis*,^44^ which corresponds to the findings of this present work against *E. coli*.

MMV1578884 (CRS-3123) has passed through a multiple-ascending-dose, single-center, randomized, placebo-controlled, double-blind phase 1 study against *Clostridioides difficile*, inhibiting type 1 methionyl-tRNA synthetase essential for protein translation.^45^ Here, CRS-3123 (PRB) presented the lowest MIC (0.019 μM), CIMB_50_ (0.0095 μM), and IC_50_ (0.019 μM) values against *S. aureus* ATCC, even lower than the antibiotic vancomycin. According to Brahma et al., the antimalarial MMV1795508 (GHPB) demonstrated significant biofilm inhibition of *S. aureus* ATCC 33592, with a higher percentage of inhibition against mature (66.48%) than preformed biofilm (less than 50%) at 10 μM.^36^

The significant stage for biofilm formation is the adherence to the surfaces leading to accumulation, colonization, and finally biofilm production; consequently, the highest prevalence of virulence factors belongs to genes involved in attachment and colonization,^46^ which justifies why not all bacteria form biofilm in the same intensity, as the *E. coli* ATCC. Kwasi and collaborators considered biofilm formation of an enteroaggregative *E. coli* 042, isolated in Peru, under 0.6 absorbance at 570 nm, inhibited by MMV688990 up to more than 80% at 5 μM.^47^

The cytotoxicity assay was performed at concentrations higher than those considered promising. MMV1580854 (PRB), which presented the lowest CIMB_50_ (2.5 μM) and IC_50_ (2.30 μM) values against *E. coli* CI, also presented satisfactory cell viability results, above 100%, which suggests its safety for the treatment of this biofilm. Ajetunmobi and collaborators demonstrated that, at 6.564 μM, MMV1593537 (PRB) reduced human hepatocellular carcinoma (HepG2) cell viability by 50% (CC_50_). In this study, the same compound demonstrated >100% viable HFF cells and 6.13% viability at 20 μM.^48^

The compounds with the lowest percentages of cell viability were from GHPB: the antituberculosis MMV1828776 with 5.06% and the antimalarial MMV1795508 with 3.98%, both at 20 μM. Moura and Reimão obtained CC_50_ at 2.77 and 6.80 μM for the same antituberculosis and antimalarial, respectively, also with HFF cells.^49^ Duffy et al. classified the mechanism of action of MMV1795508, with imidazo­[4,5 *c*]­pyridine-2,6-diamine, as chloroquine like and MMV1828776, with a pyrido­[2,3-*d*]­pyrimidine-2,4-diamine, as fast ring-stage artemisinin like against *Plasmodium falciparum* 3D7 (MRA-102).^50^

The exposure duration (24 h) selected for cytotoxicity assessment represents an early time point for evaluating compound-induced effects. Under the standard culture conditions (5% FBS, 37 °C and 5% CO_2_), HFF exhibited an average cell cycle duration of approximately 24 h, which allowed the detection of early cytotoxic effects within this interval. Nonetheless, it cannot be excluded that longer exposure times may reveal additional effects, particularly those of a predominantly cytostatic nature or with delayed onset. In the present study, several compounds elicited pronounced cytotoxicity within 24 h, resulting in substantial reductions in cell viability, indicating that this time point was sufficient to detect biologically relevant effects under the conditions tested. However, further investigations employing extended incubation periods are warranted to enable a more comprehensive characterization of toxicity profiles, including time-dependent responses and potential alterations in cell proliferation dynamics.

The most frequent interactions between bacteria and compounds were in lipid regions. MMV027339 (flucofuron) and MMV1634081 (fenoxacrim) are chemically known as pesticides/insecticides and presented the lowest CIMB_50_ and IC_50_ values from GHPB. ATR-FTIR spectral analysis revealed that flucofuron interacted with the *S. aureus* ATCC membrane, while with *S. aureus* CI interacted only with protein structures; fenoxacrim interacted with membrane and protein structures of both *S. aureus* ATCC and CI. Bethencourt-Estrella and collaborators observed that flucofuron induced plasma membrane permeability of parasites *Trypanosoma cruzi* (Y strain) and *Leishmania amazonensis* (MHOM/BR/77/LTB0016), which also contain lipids and proteins, as well as chromatin condensation, reactive oxygen species accumulation, alterations in ATP levels, and mitochondrial membrane potential.^51^

The investigation of the underlying mechanism of action for the development of effective therapeutic molecules can be assessed through molecular docking simulations.^52,53^ In this study, we observed that MMV1634081 (fenoxacrim) and MMV027339 (flucofuron) may possibly bind to the active site of *S. aureus* metalloproteinase aureolysin, which plays an essential role in immune evasion, infection, and colonization of human tissues.^54,55^ Moreover, results suggested that MMV1578884 (CRS-3123) binds to the active site of *S. aureus* nitric oxide synthase oxygenase, which is pointed out as a prominent target, given its involvement in the defense mechanism against oxidative stress.^56^ In parallel, we verified that MMV1580854 can probably bind to the active site of *E. coli* asparagine synthetase, which is involved in the synthesis of l-asparagine.^57^

On the basis of the literature, few studies have evaluated the antibacterial activity of such a large number of compounds, more than 600, some of which showed results lower than conventional antibiotics. It was the first time that the antistaphylococcal activity of the pesticides/insecticides flucofuron and fenoxacrim was demonstrated. Although the exact antibacterial mechanism of action has not been fully addressed, the results provided new and valuable insights into the investigation of effective molecules against *S. aureus* and *E. coli*. As limitations of this study of this scale, it can be pointed out that the interpretations of resazurin are only visual and, consequently, there is a lack of an analysis of the degrees of separation between the positive and negative controls.

## 5 Conclusion

Since the development of new drugs is expensive and can take years, the present study demonstrated that drugs already available on the market or in more advanced clinical stages can be repositioned between bacteria and even between bacteria and parasites or insects, for example. MMV1578884 (CRS-3123), MMV027339 (Flucofuron), and MMV1634081 (Fenoxacrim) against *S. aureus* and MMV1580854 against *E. coli* stood out for their antibacterial and antibiofilm activities, suggesting interactions with lipids and proteins and maintaining the cellular viability of human cells, which inspires the drug repositioning for these multidrug-resistant strains. Further studies are needed to better understand the mechanisms of action of the compounds, their toxicity in other cell lines, and their pharmacokinetics and pharmacodynamics in a systemic approach.

## Supplementary Material



## Notes

Minimum inhibitory concentrations (MICs) in μM for all bacteria and compounds (Table S1); Full-range ATR-FTIR spectra for results considered promising (Figures S1–S4) (PDF)
