# Supplementary material for: Drug Repositioning for Multiresistant Staphylococcus aureus and Escherichia coli from Pandemic Response Box and Global Health Priority Box of MMV
Source: ACS Omega. 2026 Aug 12;11(33):48959–71. doi: 10.1021/acsomega.5c12871 (PMC13520533; doi:10.1021/acsomega.5c12871)
Supplement: Supplementary file 1 [file ao5c12871_si_001.pdf]

**Drug repositioning for multi-resistant *Staphylococcus aureus* and *Escherichia coli* from Pandemic Response Box and Global Health Priority Box of MMV**

Daniela S. Gonçalves[1], Nagela B. S. Silva[1], Anna L. O. Santos[1], Sara L. de Souza[1], Gabriel G. Calefi[1], Ralciane de P. Menezes[1], Andreia M. de Oliveira[1], Ghabriel Honório-Silva[2], Camila M. de Andrade[2], Marcos P. O. Almeida[3], Thales A. M. Fernandes[4], Bellisa de F. Barbosa[3], Eloísa A. V. Ferro[3], Robinson Sabino-Silva[2], Carlos H. G. Martins[1*]

[1]Laboratory of Antimicrobial Testing, Institute of Biomedical Sciences, Federal University of Uberlândia, Uberlândia, Minas Gerais 38400-694, Brazil.

[2]Innovation Center in Salivary Diagnostic and Nanobiotechnology, Institute of Biomedical Sciences, Federal University of Uberlândia, Uberlândia, Minas Gerais 38400-694, Brazil.

[3]Laboratory of Immunophysiology of Reproduction, Institute of Biomedical Sciences, Federal University of Uberlândia, Uberlândia, Minas Gerais 38400-694, Brazil.

[4]Laboratory of Applied Toxinology, Butantan Institute, São Paulo, São Paulo 05503-900, Brazil.

*Corresponding author: Carlos H. G. Martins – carlos.martins2@ufu.br

Av. Pará, 1720 Block 2B Room 221

Campus Umuarama – Mailbox 592

Uberlândia - MG – 38400-694

Tel.: + 55 34 3225-8670

**Table S1.** Minimum Inhibitory Concentrations (MIC) in µM of PRB and GHPB compounds against *Staphylococcus aureus* and *Escherichia coli*.

| Compound ID | *Staphylococcus aureus* ATCC BAA-44 | *Staphylococcus aureus* clinical isolated | *Escherichia coli* ATCC BAA-198 | *Escherichia coli* clinical isolated |
|---|---|---|---|---|
| Pandemic Response Box | | | | |
| MMV1634492 | >20 | 5 | >20 | >20 |
| MMV1782108 | >20 | >20 | >20 | >20 |
| MMV1634494 | >20 | >20 | >20 | >20 |
| MMV1634386 | >20 | >20 | >20 | >20 |
| MMV637528 | >20 | >20 | >20 | >20 |
| MMV1782109 | >20 | >20 | >20 | >20 |
| MMV1634491 | >20 | >20 | >20 | >20 |
| MMV002731 | >20 | >20 | >20 | >20 |
| MMV000059 | >20 | >20 | >20 | >20 |
| MMV637104 | >20 | >20 | >20 | >20 |
| MMV637533 | >20 | 10 | ≤0.0095 | >20 |
| MMV1634362 | >20 | >20 | >20 | >20 |
| MMV1634360 | >20 | >20 | >20 | >20 |
| MMV002565 | >20 | >20 | >20 | >20 |
| MMV396785 | >20 | 2.5 | 0.31 | >20 |
| MMV003143 | >20 | >20 | >20 | 2.5 |
| MMV002337 | >20 | >20 | >20 | >20 |
| MMV1634363 | >20 | >20 | >20 | >20 |
| MMV1634359 | >20 | >20 | >20 | >20 |
| MMV1782110 | >20 | >20 | >20 | >20 |
| MMV1634493 | >20 | >20 | >20 | >20 |
| MMV344625 | >20 | >20 | >20 | >20 |
| MMV1634358 | >20 | >20 | >20 | >20 |
| MMV1578560 | 5 | >20 | >20 | >20 |
| MMV640014 | >20 | >20 | >20 | >20 |
| MMV004508 | >20 | >20 | >20 | >20 |

**Table 1.** Continued.

| Compound ID | *Staphylococcus aureus* ATCC BAA-44 | *Staphylococcus aureus* clinical isolated | *Escherichia coli* ATCC BAA-198 | *Escherichia coli* clinical isolated |
|---|---|---|---|---|
| MMV1782212 | >20 | >20 | >20 | >20 |
| MMV1782227 | >20 | >20 | >20 | >20 |
| MMV1782226 | >20 | >20 | >20 | >20 |
| MMV1782221 | >20 | >20 | >20 | >20 |
| MMV1782224 | >20 | >20 | >20 | >20 |
| MMV1782223 | >20 | >20 | >20 | >20 |
| MMV1782218 | >20 | >20 | >20 | >20 |
| MMV1782217 | >20 | >20 | >20 | >20 |
| MMV1782216 | >20 | >20 | >20 | >20 |
| MMV1009286 | >20 | >20 | ≤0.0095 | >20 |
| MMV1782225 | >20 | >20 | >20 | >20 |
| MMV1782354 | >20 | >20 | >20 | >20 |
| MMV689401 | >20 | >20 | >20 | >20 |
| MMV1782351 | >20 | >20 | >20 | >20 |
| MMV1782387 | >20 | >20 | >20 | >20 |
| MMV1782389 | >20 | >20 | >20 | >20 |
| MMV1782388 | >20 | >20 | >20 | >20 |
| MMV1634557 | >20 | >20 | >20 | >20 |
| MMV030787 | >20 | >20 | >20 | >20 |
| MMV002722 | >20 | >20 | >20 | >20 |
| MMV1593535 | >20 | >20 | >20 | >20 |
| MMV1593533 | >20 | >20 | >20 | >20 |
| MMV422940 | >20 | >20 | 20 | >20 |
| MMV1582497 | 20 | 20 | >20 | >20 |
| MMV1581553 | >20 | 5 | >20 | >20 |
| MMV1581548 | 20 | ≤0.0095 | 20 | >20 |
| MMV1580850 | 20 | >20 | >20 | >20 |

**Table 1.** Continued.

| Compound ID | *Staphylococcus aureus* ATCC BAA-44 | *Staphylococcus aureus* clinical isolated | *Escherichia coli* ATCC BAA-198 | *Escherichia coli* clinical isolated |
|---|---|---|---|---|
| MMV1579783 | 20 | ≤0.0095 | >20 | >20 |
| MMV1578925 | 20 | >20 | >20 | >20 |
| MMV1578889 | 20 | 20 | 20 | >20 |
| MMV1578572 | >20 | >20 | >20 | >20 |
| MMV1580173 | 5 | 2.5 | 0.15 | >20 |
| MMV1634403 | >20 | >20 | >20 | >20 |
| MMV1634390 | ≤0.0095 | ≤0.0095 | >20 | >20 |
| MMV1633967 | 10 | ≤0.0095 | >20 | >20 |
| MMV1593534 | 20 | >20 | >20 | >20 |
| MMV1582498 | >20 | >20 | >20 | >20 |
| MMV1582494 | >20 | >20 | >20 | >20 |
| MMV1582493 | >20 | >20 | >20 | >20 |
| MMV000043 | >20 | >20 | >20 | >20 |
| MMV1581552 | >20 | >20 | >20 | >20 |
| MMV1581551 | >20 | 20 | >20 | >20 |
| MMV1580853 | 0.31 | 1.25 | 1.25 | >20 |
| MMV1580841 | 20 | 20 | 0.078 | >20 |
| MMV1579878 | >20 | >20 | >20 | >20 |
| MMV1579845 | >20 | >20 | >20 | >20 |
| MMV1579787 | >20 | >20 | >20 | >20 |
| MMV1579786 | >20 | >20 | >20 | >20 |
| MMV1579784 | >20 | >20 | >20 | >20 |
| MMV1483032 | >20 | >20 | >20 | >20 |
| MMV508427 | >20 | >20 | >20 | >20 |
| MMV1578565 | >20 | >20 | >20 | >20 |
| MMV1578561 | 20 | >20 | >20 | >20 |
| MMV1578557 | 10 | 1.25 | >20 | >20 |

**Table 1.** Continued.

| Compound ID | *Staphylococcus aureus* ATCC BAA-44 | *Staphylococcus aureus* clinical isolated | *Escherichia coli* ATCC BAA-198 | *Escherichia coli* clinical isolated |
|---|---|---|---|---|
| MMV000725 | 20 | 20 | 20 | >20 |
| MMV1634384 | >20 | >20 | >20 | >20 |
| MMV1634383 | >20 | >20 | >20 | >20 |
| MMV1613560 | >20 | 20 | >20 | >20 |
| MMV1230557 | >20 | >20 | >20 | >20 |
| MMV1593540 | >20 | >20 | >20 | >20 |
| MMV1593539 | 10 | 10 | >20 | >20 |
| MMV1582495 | >20 | >20 | >20 | >20 |
| MMV1582487 | >20 | >20 | >20 | >20 |
| MMV021759 | >20 | >20 | >20 | >20 |
| MMV1581555 | >20 | >20 | >20 | >20 |
| MMV1581547 | >20 | 0.31 | >20 | >20 |
| MMV687800 | >20 | 2.5 | >20 | >20 |
| MMV1580848 | >20 | 10 | >20 | >20 |
| MMV1580842 | >20 | 20 | >20 | >20 |
| MMV1580840 | >20 | 20 | >20 | >20 |
| MMV046261 | >20 | 20 | >20 | >20 |
| MMV1579846 | >20 | 20 | 1.25 | >20 |
| MMV1578842 | >20 | >20 | >20 | >20 |
| MMV1579776 | >20 | >20 | >20 | >20 |
| MMV1579775 | >20 | >20 | >20 | >20 |
| MMV1579354 | >20 | >20 | >20 | >20 |
| MMV1578899 | >20 | >20 | >20 | >20 |
| MMV1578890 | >20 | 20 | >20 | >20 |
| MMV1578886 | >20 | >20 | >20 | >20 |
| MMV1578573 | >20 | >20 | >20 | >20 |
| MMV1578571 | >20 | 20 | >20 | >20 |

**Table 1.** Continued.

| Compound ID | *Staphylococcus aureus* ATCC BAA-44 | *Staphylococcus aureus* clinical isolated | *Escherichia coli* ATCC BAA-198 | *Escherichia coli* clinical isolated |
|---|---|---|---|---|
| MMV687273 | >20 | 5 | >20 | >20 |
| MMV1634387 | >20 | >20 | >20 | ≤0.0095 |
| MMV1633966 | >20 | 2.5 | >20 | >20 |
| MMV1505642 | >20 | >20 | >20 | >20 |
| MMV1593542 | >20 | >20 | >20 | >20 |
| MMV1593538 | >20 | >20 | >20 | >20 |
| MMV002459 | >20 | >20 | 5 | >20 |
| MMV1580854 | >20 | 0.15 | 0.62 | 0.62 |
| MMV1580851 | >20 | >20 | >20 | >20 |
| MMV1579844 | >20 | 20 | >20 | >20 |
| MMV141011 | >20 | >20 | >20 | >20 |
| MMV002516 | >20 | >20 | >20 | >20 |
| MMV688755 | >20 | >20 | >20 | >20 |
| MMV020752 | 0.019 | 5 | >20 | >20 |
| MMV1229204 | 1.25 | >20 | >20 | >20 |
| MMV1578575 | 0.31 | 1.25 | >20 | >20 |
| MMV1578556 | 1.25 | >20 | >20 | >20 |
| MMV1663457 | >20 | ≤0.0095 | >20 | >20 |
| MMV1341773 | >20 | 0.039 | >20 | >20 |
| MMV1633970 | >20 | >20 | >20 | >20 |
| MMV1633965 | 0.31 | 20 | >20 | >20 |
| MMV093250 | 5 | >20 | >20 | >20 |
| MMV1593541 | 1.25 | 0.62 | >20 | >20 |
| MMV1593537 | 5 | 1.25 | >20 | >20 |
| MMV1582492 | >20 | >20 | >20 | >20 |
| MMV1582491 | >20 | >20 | >20 | 0.31 |
| MMV1582382 | >20 | ≤0.0095 | >20 | >20 |

**Table 1.** Continued.

| Compound ID | *Staphylococcus aureus* ATCC BAA-44 | *Staphylococcus aureus* clinical isolated | *Escherichia coli* ATCC BAA-198 | *Escherichia coli* clinical isolated |
|---|---|---|---|---|
| MMV1581557 | >20 | >20 | >20 | >20 |
| MMV1581554 | >20 | >20 | >20 | >20 |
| MMV1581550 | >20 | >20 | >20 | >20 |
| MMV1581546 | >20 | >20 | >20 | >20 |
| MMV1580844 | >20 | >20 | >20 | >20 |
| MMV1579788 | >20 | ≤0.0095 | >20 | >20 |
| MMV1579785 | >20 | ≤0.0095 | >20 | >20 |
| MMV811071 | >20 | >20 | >20 | >20 |
| MMV1578898 | >20 | >20 | >20 | >20 |
| MMV1549626 | >20 | >20 | >20 | >20 |
| MMV1578579 | ≤0.0095 | 20 | >20 | >20 |
| MMV1578578 | >20 | >20 | >20 | >20 |
| MMV1578569 | >20 | >20 | >20 | >20 |
| MMV1578566 | >20 | 5 | 2.5 | 1.25 |
| MMV1578563 | >20 | >20 | >20 | >20 |
| MMV1578558 | >20 | >20 | >20 | >20 |
| MMV1782140 | 20 | >20 | >20 | >20 |
| MMV1634391 | 2.5 | 5 | >20 | >20 |
| MMV1633674 | ≤0.0095 | 0.019 | 1.25 | 2.5 |
| MMV002169 | >20 | >20 | >20 | >20 |
| MMV002612 | >20 | >20 | 1.25 | 5 |
| MMV1613562 | >20 | >20 | >20 | >20 |
| MMV689758 | >20 | >20 | >20 | >20 |
| MMV1593532 | >20 | >20 | >20 | >20 |
| MMV637659 | 5 | >20 | 0.31 | >20 |
| MMV1580846 | >20 | >20 | >20 | >20 |
| MMV1580845 | >20 | >20 | >20 | >20 |

**Table 1.** Continued.

| Compound ID | *Staphylococcus aureus* ATCC BAA-44 | *Staphylococcus aureus* clinical isolated | *Escherichia coli* ATCC BAA-198 | *Escherichia coli* clinical isolated |
|---|---|---|---|---|
| MMV1579850 | 1.25 | >20 | 0.019 | 5 |
| MMV374187 | >20 | >20 | >20 | >20 |
| MMV1579781 | >20 | >20 | >20 | >20 |
| MMV1579780 | >20 | >20 | >20 | >20 |
| MMV688756 | 1.25 | 2.5 | >20 | >20 |
| MMV1578897 | 20 | 20 | >20 | >20 |
| MMV001014 | >20 | >20 | >20 | >20 |
| MMV1578885 | >20 | >20 | >20 | >20 |
| MMV687696 | >20 | >20 | >20 | >20 |
| MMV011565 | >20 | >20 | >20 | >20 |
| MMV452821 | >20 | >20 | >20 | >20 |
| MMV1578576 | >20 | >20 | >20 | >20 |
| MMV1578562 | >20 | >20 | >20 | >20 |
| MMV1578555 | >20 | >20 | >20 | >20 |
| MMV1578554 | >20 | >20 | >20 | >20 |
| MMV1634399 | >20 | >20 | >20 | >20 |
| MMV002287 | >20 | >20 | >20 | >20 |
| MMV831201 | >20 | >20 | >20 | >20 |
| MMV687801 | >20 | >20 | >20 | >20 |
| MMV1593531 | >20 | >20 | >20 | >20 |
| MMV1581558 | >20 | >20 | >20 | >20 |
| MMV1581556 | >20 | >20 | >20 | >20 |
| MMV1581549 | 20 | 20 | 1.25 | >20 |
| MMV1580847 | >20 | 20 | >20 | >20 |
| MMV1579847 | >20 | >20 | >20 | >20 |
| MMV1579782 | >20 | >20 | >20 | >20 |
| MMV1579777 | >20 | >20 | >20 | >20 |

**Table 1.** Continued.

| Compound ID | *Staphylococcus aureus* ATCC BAA-44 | *Staphylococcus aureus* clinical isolated | *Escherichia coli* ATCC BAA-198 | *Escherichia coli* clinical isolated |
|---|---|---|---|---|
| MMV292173 | >20 | >20 | >20 | >20 |
| MMV102270 | >20 | >20 | >20 | >20 |
| MMV00008 | >20 | >20 | >20 | >20 |
| MMV002260 | >20 | >20 | >20 | >20 |
| MMV102833 | 10 | 20 | >20 | >20 |
| MMV565773 | >20 | >20 | >20 | >20 |
| MMV1578577 | >20 | >20 | >20 | >20 |
| MMV233495 | >20 | >20 | >20 | >20 |
| MMV002676 | >20 | >20 | >20 | >20 |
| MMV1633675 | >20 | >20 | >20 | >20 |
| MMV303733 | >20 | >20 | >20 | >20 |
| MMV003249 | >20 | >20 | >20 | >20 |
| MMV001438 | >20 | >20 | >20 | >20 |
| MMV1582496 | 10 | 5 | >20 | >20 |
| MMV1582490 | >20 | 20 | 20 | >20 |
| MMV1582489 | >20 | >20 | >20 | >20 |
| MMV1582488 | >20 | >20 | >20 | >20 |
| MMV1581559 | >20 | >20 | >20 | >20 |
| MMV1581545 | 20 | 20 | >20 | >20 |
| MMV1580855 | >20 | >20 | >20 | >20 |
| MMV1580852 | 20 | >20 | >20 | >20 |
| MMV1580849 | 2.5 | 1.25 | 0.078 | >20 |
| MMV1580839 | >20 | >20 | >20 | >20 |
| MMV1579779 | >20 | >20 | >20 | >20 |
| MMV1579778 | >20 | >20 | >20 | >20 |
| MMV099714 | >20 | >20 | >20 | >20 |
| MMV1578891 | 2.5 | >20 | >20 | >20 |

**Table 1.** Continued.

| Compound ID | *Staphylococcus aureus* ATCC BAA-44 | *Staphylococcus aureus* clinical isolated | *Escherichia coli* ATCC BAA-198 | *Escherichia coli* clinical isolated |
|---|---|---|---|---|
| MMV124656 | >20 | >20 | >20 | >20 |
| MMV975972 | >20 | >20 | >20 | >20 |
| MMV1578884 | 0.019 | 0.078 | >20 | >20 |
| MMV108465 | >20 | >20 | >20 | >20 |
| MMV1480967 | >20 | >20 | 0.062 | >20 |
| MMV1578570 | 10 | 10 | 20 | 20 |
| MMV1782215 | >20 | >20 | >20 | >20 |
| MMV1782403 | 1.25 | 1.25 | >20 | >20 |
| MMV1782402 | >20 | >20 | >20 | >20 |
| MMV1782411 | >20 | >20 | >20 | >20 |
| MMV214956 | >20 | >20 | >20 | >20 |
| MMV1634395 | >20 | >20 | >20 | >20 |
| MMV1634071 | >20 | >20 | >20 | >20 |
| MMV1633962 | >20 | >20 | >20 | >20 |
| MMV1633678 | >20 | >20 | >20 | >20 |
| MMV690480 | >20 | >20 | >20 | >20 |
| MMV1593517 | >20 | >20 | >20 | >20 |
| MMV1593515 | >20 | >20 | >20 | >20 |
| MMV1581032 | 20 | 10 | >20 | >20 |
| MMV1580799 | >20 | >20 | >20 | >20 |
| MMV001726 | >20 | >20 | >20 | >20 |
| MMV690653 | >20 | >20 | >20 | >20 |
| MMV1580484 | >20 | >20 | >20 | >20 |
| MMV1580478 | >20 | >20 | >20 | >20 |
| MMV1782102 | >20 | >20 | >20 | >20 |
| MMV394033 | 10 | 20 | 20 | >20 |
| MMV1634404 | >20 | >20 | >20 | >20 |

**Table 1.** Continued.

| Compound ID | *Staphylococcus aureus* ATCC BAA-44 | *Staphylococcus aureus* clinical isolated | *Escherichia coli* ATCC BAA-198 | *Escherichia coli* clinical isolated |
|---|---|---|---|---|
| MMV1634388 | >20 | >20 | >20 | >20 |
| MMV180402 | >20 | >20 | >20 | >20 |
| MMV642550 | >20 | >20 | >20 | >20 |
| MMV1580798 | >20 | >20 | >20 | >20 |
| MMV1580800 | >20 | >20 | >20 | >20 |
| MMV1580503 | >20 | >20 | >20 | >20 |
| MMV690547 | >20 | >20 | >20 | >20 |
| MMV1165877 | >20 | >20 | >20 | >20 |
| MMV1580497 | >20 | >20 | >20 | >20 |
| MMV1580495 | >20 | >20 | >20 | >20 |
| MMV1645152 | >20 | >20 | >20 | >20 |
| MMV1633677 | >20 | >20 | >20 | >20 |
| MMV1593520 | >20 | >20 | >20 | >20 |
| MMV1581029 | >20 | >20 | >20 | >20 |
| MMV1580794 | >20 | >20 | >20 | >20 |
| MMV001793 | >20 | >20 | >20 | >20 |
| MMV251679 | >20 | >20 | >20 | >20 |
| MMV003291 | >20 | >20 | >20 | >20 |
| MMV009948 | >20 | >20 | >20 | >20 |
| MMV1174026 | >20 | >20 | >20 | >20 |
| MMV003820 | >20 | >20 | >20 | >20 |
| MMV639951 | >20 | >20 | >20 | >20 |
| MMV1580491 | >20 | >20 | >20 | >20 |
| MMV1580492 | 20 | 20 | >20 | >20 |
| MMV690621 | >20 | >20 | >20 | >20 |
| MMV1782112 | >20 | >20 | >20 | >20 |
| MMV1782107 | >20 | >20 | >20 | >20 |

**Table 1.** Continued.

| Compound ID | *Staphylococcus aureus* ATCC BAA-44 | *Staphylococcus aureus* clinical isolated | *Escherichia coli* ATCC BAA-198 | *Escherichia coli* clinical isolated |
|---|---|---|---|---|
| MMV1782106 | >20 | >20 | >20 | >20 |
| MMV1782105 | >20 | >20 | >20 | >20 |
| MMV1782104 | >20 | >20 | >20 | >20 |
| MMV1782101 | >20 | >20 | >20 | >20 |
| MMV1633969 | >20 | >20 | >20 | >20 |
| MMV596723 | >20 | >20 | >20 | >20 |
| MMV1581378 | >20 | >20 | >20 | >20 |
| MMV1581031 | >20 | >20 | >20 | >20 |
| MMV1580797 | >20 | >20 | >20 | >20 |
| MMV001761 | >20 | >20 | >20 | >20 |
| MMV002015 | >20 | >20 | >20 | >20 |
| MMV056052 | >20 | >20 | >20 | >20 |
| MMV1580504 | >20 | >20 | >20 | >20 |
| MMV001961 | >20 | >20 | >20 | >20 |
| MMV003738 | >20 | >20 | >20 | >20 |
| MMV690540 | >20 | >20 | >20 | >20 |
| MMV614278 | >20 | >20 | >20 | >20 |
| MMV1580489 | 20 | 10 | >20 | >20 |
| MMV1580499 | >20 | >20 | >20 | >20 |
| MMV1782115 | >20 | >20 | >20 | >20 |
| MMV1782114 | >20 | >20 | >20 | >20 |
| MMV018362 | >20 | >20 | >20 | >20 |
| MMV1634556 | >20 | >20 | >20 | >20 |
| MMV1634401 | >20 | >20 | >20 | >20 |
| MMV1634394 | >20 | >20 | >20 | >20 |
| MMV1633964 | >20 | >20 | >20 | >20 |
| MMV1593516 | >20 | >20 | >20 | >20 |

**Table 1.** Continued.

| Compound ID | *Staphylococcus aureus* ATCC BAA-44 | *Staphylococcus aureus* clinical isolated | *Escherichia coli* ATCC BAA-198 | *Escherichia coli* clinical isolated |
|---|---|---|---|---|
| MMV1593513 | >20 | >20 | >20 | >20 |
| MMV1593511 | >20 | >20 | >20 | >20 |
| MMV637306 | >20 | >20 | >20 | >20 |
| MMV1581377 | >20 | >20 | >20 | >20 |
| MMV002780 | >20 | >20 | >20 | >20 |
| MMV1580801 | ≤0.0095 | >20 | >20 | >20 |
| MMV010036 | >20 | >20 | >20 | >20 |
| MMV637879 | >20 | >20 | >20 | >20 |
| MMV617332 | >20 | >20 | >20 | >20 |
| MMV1580480 | >20 | >20 | >20 | >20 |
| MMV1580494 | >20 | >20 | >20 | >20 |
| MMV275100 | >20 | >20 | >20 | >20 |
| MMV1782103 | >20 | >20 | >20 | >20 |
| MMV1782098 | >20 | >20 | >20 | >20 |
| MMV1634397 | >20 | >20 | >20 | >20 |
| MMV1633963 | >20 | >20 | >20 | >20 |
| MMV1593519 | >20 | >20 | >20 | >20 |
| MMV002505 | 20 | 20 | >20 | >20 |
| MMV1581034 | >20 | >20 | >20 | >20 |
| MMV1581033 | >20 | >20 | >20 | >20 |
| MMV1557856 | >20 | >20 | >20 | >20 |
| MMV1580796 | >20 | 20 | >20 | >20 |
| MMV1580505 | >20 | >20 | >20 | >20 |
| MMV003297 | >20 | >20 | >20 | >20 |
| MMV1580487 | >20 | >20 | >20 | >20 |
| MMV1580485 | >20 | >20 | >20 | >20 |
| MMV098836 | >20 | >20 | >20 | >20 |

**Table 1.** Continued.

| Compound ID | *Staphylococcus aureus* ATCC BAA-44 | *Staphylococcus aureus* clinical isolated | *Escherichia coli* ATCC BAA-198 | *Escherichia coli* clinical isolated |
|---|---|---|---|---|
| MMV218827 | >20 | >20 | >20 | >20 |
| MMV1782111 | >20 | >20 | >20 | >20 |
| MMV1782097 | >20 | >20 | >20 | >20 |
| MMV1634398 | >20 | >20 | >20 | >20 |
| MMV1634396 | >20 | >20 | >20 | >20 |
| MMV1634385 | 10 | 20 | >20 | >20 |
| MMV1633968 | >20 | >20 | >20 | >20 |
| MMV688991 | >20 | >20 | >20 | >20 |
| MMV1580502 | >20 | >20 | >20 | >20 |
| MMV637855 | >20 | >20 | >20 | >20 |
| MMV690467 | >20 | >20 | >20 | >20 |
| MMV1580493 | >20 | >20 | >20 | >20 |
| MMV1580482 | 10 | 10 | >20 | >20 |
| MMV1580501 | >20 | >20 | >20 | >20 |
| MMV1580496 | >20 | >20 | >20 | >20 |
| MMV1580490 | >20 | >20 | >20 | >20 |
| MMV1782113 | >20 | >20 | >20 | >20 |
| MMV1634393 | 20 | >20 | >20 | >20 |
| MMV1634392 | >20 | >20 | >20 | >20 |
| MMV1634389 | >20 | >20 | >20 | >20 |
| MMV1581035 | >20 | >20 | >20 | >20 |
| MMV1593544 | >20 | >20 | >20 | >20 |
| MMV1593521 | >20 | >20 | >20 | >20 |
| MMV1593514 | >20 | >20 | >20 | >20 |
| MMV1581036 | >20 | >20 | >20 | >20 |
| MMV1581030 | >20 | >20 | >20 | >20 |
| MMV247764 | >20 | >20 | >20 | >20 |

**Table 1.** Continued.

| Compound ID | *Staphylococcus aureus* ATCC BAA-44 | *Staphylococcus aureus* clinical isolated | *Escherichia coli* ATCC BAA-198 | *Escherichia coli* clinical isolated |
|---|---|---|---|---|
| MMV019724 | >20 | >20 | >20 | >20 |
| MMV638198 | >20 | >20 | >20 | >20 |
| MMV637413 | 2.5 | 10 | 2.5 | >20 |
| MMV658803 | 10 | 10 | >20 | >20 |
| MMV1580500 | >20 | >20 | >20 | >20 |
| MMV1580498 | >20 | >20 | >20 | >20 |
| MMV1580488 | >20 | >20 | >20 | >20 |
| MMV1580486 | >20 | >20 | >20 | >20 |
| MMV1782222 | >20 | >20 | >20 | >20 |
| MMV690706 | >20 | >20 | >20 | >20 |
| MMV1782220 | >20 | >20 | >20 | >20 |
| MMV1782213 | >20 | >20 | >20 | >20 |
| MMV1782214 | >20 | >20 | >20 | >20 |
| MMV1782211 | >20 | 5 | >20 | >20 |
| MMV1782210 | >20 | >20 | >20 | >20 |
| MMV1782208 | >20 | >20 | >20 | >20 |
| MMV1006203 | >20 | >20 | >20 | >20 |
| MMV1782353 | >20 | >20 | >20 | >20 |
| MMV1782349 | >20 | >20 | >20 | >20 |
| MMV1782355 | >20 | >20 | >20 | >20 |
| MMV1782352 | >20 | >20 | >20 | >20 |
| MMV1782350 | >20 | >20 | >20 | >20 |
| MMV1782386 | >20 | >20 | >20 | >20 |
| MMV1782412 | >20 | >20 | >20 | >20 |
| MMV1580843 | 20 | >20 | >20 | >20 |
| MMV690555 | >20 | >20 | >20 | >20 |
| MMV1613559 | >20 | >20 | >20 | >20 |

**Table 1.** Continued.

| Compound ID | *Staphylococcus aureus* ATCC BAA-44 | *Staphylococcus aureus* clinical isolated | *Escherichia coli* ATCC BAA-198 | *Escherichia coli* clinical isolated |
|---|---|---|---|---|
| MMV1634361 | >20 | >20 | >20 | 5 |
| MMV1580483 | >20 | >20 | >20 | >20 |
| MMV1782209 | >20 | >20 | >20 | >20 |
| MMV003069 | >20 | >20 | >20 | >20 |
| Global Health Priority Box | | | | |
| MMV1793194 | >20 | >20 | >20 | >20 |
| MMV1794212 | >20 | >20 | >20 | >20 |
| MMV1794213 | >20 | >20 | >20 | >20 |
| MMV1794214 | >20 | >20 | >20 | >20 |
| MMV1794211 | >20 | >20 | >20 | >20 |
| MMV1794207 | >20 | >20 | >20 | >20 |
| MMV002287 | >20 | >20 | >20 | >20 |
| MMV673126 | >20 | >20 | >20 | >20 |
| MMV1578924 | 20 | 20 | >20 | >20 |
| MMV002371 | >20 | >20 | >20 | >20 |
| MMV083774 | >20 | >20 | >20 | >20 |
| MMV637085 | >20 | >20 | >20 | >20 |
| MMV1634083 | >20 | >20 | >20 | >20 |
| MMV1633828 | 20 | 20 | >20 | >20 |
| MMV689586 | 20 | 20 | >20 | >20 |
| MMV1796254 | >20 | >20 | >20 | >20 |
| MMV1633823 | >20 | >20 | >20 | >20 |
| MMV1634080 | >20 | >20 | >20 | >20 |
| MMV002519 | >20 | >20 | >20 | >20 |
| MMV003977 | >20 | >20 | >20 | >20 |
| MMV1634506 | >20 | >20 | >20 | >20 |
| MMV1577469 | >20 | >20 | >20 | >20 |

**Table 1.** Continued.

| Compound ID | *Staphylococcus aureus* ATCC BAA-44 | *Staphylococcus aureus* clinical isolated | *Escherichia coli* ATCC BAA-198 | *Escherichia coli* clinical isolated |
|---|---|---|---|---|
| MMV1794216 | >20 | >20 | >20 | >20 |
| MMV003958 | >20 | >20 | >20 | >20 |
| MMV1577459 | >20 | >20 | >20 | >20 |
| MMV672931 | 20 | 20 | >20 | >20 |
| MMV689404 | >20 | >20 | >20 | >20 |
| MMV003972 | >20 | >20 | >20 | >20 |
| MMV1633824 | >20 | >20 | >20 | >20 |
| MMV078538 | >20 | >20 | >20 | >20 |
| MMV1633826 | >20 | >20 | >20 | >20 |
| MMV688934 | >20 | >20 | >20 | >20 |
| MMV1634082 | >20 | >20 | >20 | >20 |
| MMV637136 | >20 | >20 | >20 | >20 |
| MMV1633827 | >20 | >20 | >20 | >20 |
| MMV1577454 | 10 | 20 | >20 | >20 |
| MMV1577464 | >20 | >20 | >20 | >20 |
| MMV002231 | 10 | 20 | >20 | >20 |
| MMV1633825 | >20 | >20 | >20 | >20 |
| MMV1577472 | >20 | >20 | >20 | >20 |
| MMV1577465 | ≤0.0095 | ≤0.0095 | >20 | >20 |
| MMV689409 | >20 | >20 | >20 | >20 |
| MMV027339 | 2.5 | 2.5 | >20 | >20 |
| MMV1577453 | >20 | >20 | >20 | >20 |
| MMV1577458 | >20 | >20 | >20 | >20 |
| MMV1577463 | >20 | >20 | >20 | >20 |
| MMV1577467 | >20 | >20 | >20 | >20 |
| MMV1577466 | >20 | >20 | >20 | >20 |
| MMV1577452 | >20 | >20 | >20 | >20 |

**Table 1.** Continued.

| Compound ID | *Staphylococcus aureus* ATCC BAA-44 | *Staphylococcus aureus* clinical isolated | *Escherichia coli* ATCC BAA-198 | *Escherichia coli* clinical isolated |
|---|---|---|---|---|
| MMV003872 | >20 | >20 | >20 | >20 |
| MMV1577455 | >20 | >20 | >20 | >20 |
| MMV003891 | >20 | >20 | >20 | >20 |
| MMV1577474 | >20 | >20 | >20 | >20 |
| MMV1577471 | 20 | 20 | >20 | >20 |
| MMV1577460 | >20 | >20 | >20 | >20 |
| MMV003975 | >20 | >20 | >20 | >20 |
| MMV1634081 | 2.5 | 2.5 | >20 | >20 |
| MMV1577461 | >20 | >20 | >20 | >20 |
| MMV1633829 | >20 | >20 | >20 | >20 |
| MMV004017 | >20 | >20 | >20 | >20 |
| MMV1577890 | >20 | >20 | >20 | >20 |
| MMV1634079 | >20 | >20 | >20 | >20 |
| MMV1577456 | >20 | >20 | >20 | >20 |
| MMV1577470 | >20 | >20 | >20 | >20 |
| MMV1577889 | >20 | >20 | >20 | >20 |
| MMV689416 | >20 | >20 | >20 | >20 |
| MMV1796401 | >20 | >20 | >20 | >20 |
| MMV637769 | >20 | >20 | >20 | >20 |
| MMV1501398 | >20 | >20 | >20 | >20 |
| MMV1577462 | >20 | >20 | >20 | >20 |
| MMV1577473 | >20 | >20 | >20 | >20 |
| MMV1577468 | >20 | >20 | >20 | >20 |
| MMV1794204 | >20 | >20 | >20 | >20 |
| MMV1794205 | >20 | >20 | >20 | >20 |
| MMV1794210 | >20 | >20 | >20 | >20 |
| MMV1794215 | >20 | >20 | >20 | >20 |

**Table 1.** Continued.

| Compound ID | *Staphylococcus aureus* ATCC BAA-44 | *Staphylococcus aureus* clinical isolated | *Escherichia coli* ATCC BAA-198 | *Escherichia coli* clinical isolated |
|---|---|---|---|---|
| MMV1794209 | >20 | >20 | >20 | >20 |
| MMV1794206 | >20 | >20 | >20 | >20 |
| MMV003974 | >20 | >20 | >20 | >20 |
| MMV1634507 | >20 | >20 | >20 | >20 |
| MMV1791425 | >20 | >20 | >20 | >20 |
| MMV1726212 | >20 | >20 | >20 | >20 |
| MMV1841740 | 20 | 10 | 20 | >20 |
| MMV1752548 | >20 | >20 | >20 | >20 |
| MMV1805007 | >20 | >20 | >20 | >20 |
| MMV1704710 | >20 | >20 | >20 | >20 |
| MMV006187 | >20 | >20 | >20 | >20 |
| MMV1798946 | >20 | >20 | >20 | >20 |
| MMV020508 | >20 | >20 | >20 | >20 |
| MMV1808809 | >20 | >20 | >20 | >20 |
| MMV020335 | >20 | >20 | >20 | >20 |
| MMV1797658 | >20 | >20 | >20 | >20 |
| MMV1760745 | >20 | >20 | >20 | >20 |
| MMV1845852 | >20 | >20 | >20 | >20 |
| MMV007007 | >20 | >20 | >20 | >20 |
| MMV007282 | >20 | >20 | >20 | >20 |
| MMV023190 | >20 | >20 | >20 | >20 |
| MMV1804559 | 10 | 20 | >20 | >20 |
| MMV670631 | >20 | >20 | >20 | >20 |
| MMV006931 | >20 | >20 | >20 | >20 |
| MMV006430 | >20 | >20 | >20 | >20 |
| MMV025013 | >20 | >20 | >20 | >20 |
| MMV1167451 | >20 | >20 | >20 | >20 |

**Table 1.** Continued.

| Compound ID | *Staphylococcus aureus* ATCC BAA-44 | *Staphylococcus aureus* clinical isolated | *Escherichia coli* ATCC BAA-198 | *Escherichia coli* clinical isolated |
|---|---|---|---|---|
| MMV011471 | >20 | >20 | >20 | >20 |
| MMV007501 | >20 | >20 | >20 | >20 |
| MMV020192 | >20 | >20 | >20 | >20 |
| MMV1103183 | >20 | >20 | >20 | >20 |
| MMV978042 | >20 | >20 | >20 | >20 |
| MMV936405 | >20 | >20 | >20 | >20 |
| MMV959706 | >20 | >20 | >20 | >20 |
| MMV020207 | 10 | 5 | >20 | >20 |
| MMV001197 | >20 | >20 | >20 | >20 |
| MMV020196 | 20 | >20 | >20 | >20 |
| MMV020033 | >20 | >20 | >20 | >20 |
| MMV019547 | >20 | >20 | 5 | >20 |
| MMV026458 | >20 | >20 | >20 | >20 |
| MMV020456 | >20 | >20 | >20 | >20 |
| MMV020203 | >20 | >20 | >20 | >20 |
| MMV020709 | >20 | >20 | >20 | >20 |
| MMV023573 | 20 | >20 | >20 | >20 |
| MMV006344 | >20 | >20 | >20 | >20 |
| MMV023523 | >20 | >20 | >20 | >20 |
| MMV024638 | 20 | 20 | 20 | >20 |
| MMV1542706 | >20 | >20 | >20 | >20 |
| MMV006597 | >20 | >20 | >20 | >20 |
| MMV1795555 | >20 | >20 | >20 | >20 |
| MMV660057 | >20 | >20 | >20 | >20 |
| MMV006219 | >20 | >20 | >20 | >20 |
| MMV007300 | >20 | >20 | >20 | >20 |
| MMV028822 | >20 | >20 | >20 | >20 |

**Table 1.** Continued.

| Compound ID | *Staphylococcus aureus* ATCC BAA-44 | *Staphylococcus aureus* clinical isolated | *Escherichia coli* ATCC BAA-198 | *Escherichia coli* clinical isolated |
|---|---|---|---|---|
| MMV024850 | >20 | >20 | >20 | >20 |
| MMV023401 | >20 | >20 | >20 | >20 |
| MMV085494 | >20 | >20 | >20 | >20 |
| MMV1795557 | >20 | 20 | >20 | >20 |
| MMV1796046 | >20 | >20 | >20 | >20 |
| MMV674132 | >20 | >20 | >20 | >20 |
| MMV753519 | >20 | >20 | >20 | >20 |
| MMV981619 | >20 | >20 | >20 | >20 |
| MMV979319 | >20 | >20 | >20 | >20 |
| MMV979930 | >20 | >20 | >20 | 0.62 |
| MMV974630 | >20 | >20 | >20 | >20 |
| MMV1267536 | >20 | >20 | >20 | >20 |
| MMV1266067 | >20 | >20 | >20 | >20 |
| MMV1262756 | >20 | >20 | >20 | >20 |
| MMV019421 | 10 | 10 | >20 | >20 |
| MMV1435700 | >20 | >20 | >20 | >20 |
| MMV962005 | >20 | >20 | >20 | >20 |
| MMV027331 | >20 | >20 | >20 | >20 |
| MMV014853 | >20 | >20 | >20 | >20 |
| MMV026953 | >20 | >20 | >20 | >20 |
| MMV1804275 | >20 | >20 | >20 | >20 |
| MMV019177 | >20 | >20 | >20 | >20 |
| MMV029587 | >20 | >20 | >20 | >20 |
| MMV019053 | >20 | >20 | >20 | >20 |
| MMV023951 | >20 | >20 | >20 | >20 |
| MMV022487 | >20 | >20 | >20 | >20 |
| MMV020445 | >20 | >20 | >20 | >20 |

**Table 1.** Continued.

| Compound ID | *Staphylococcus aureus* ATCC BAA-44 | *Staphylococcus aureus* clinical isolated | *Escherichia coli* ATCC BAA-198 | *Escherichia coli* clinical isolated |
|---|---|---|---|---|
| MMV006621 | >20 | >20 | >20 | >20 |
| MMV024309 | >20 | >20 | >20 | >20 |
| MMV024825 | >20 | >20 | >20 | >20 |
| MMV673982 | >20 | >20 | >20 | >20 |
| MMV1558322 | >20 | >20 | >20 | >20 |
| MMV1558949 | >20 | >20 | >20 | >20 |
| MMV692630 | >20 | >20 | >20 | >20 |
| MMV692861 | >20 | >20 | >20 | >20 |
| MMV674787 | >20 | >20 | >20 | >20 |
| MMV1795508 | 5 | 5 | 20 | >20 |
| MMV689461 | 20 | 20 | 20 | 20 |
| MMV1542798 | 10 | 10 | 20 | >20 |
| MMV1542799 | 10 | 10 | >20 | >20 |
| MMV1542822 | >20 | >20 | >20 | >20 |
| MMV1542820 | >20 | >20 | >20 | >20 |
| MMV692107 | 20 | 20 | >20 | >20 |
| MMV1829088 | >20 | >20 | >20 | >20 |
| MMV1829377 | >20 | >20 | >20 | >20 |
| MMV1828855 | >20 | >20 | >20 | >20 |
| MMV1829150 | >20 | >20 | >20 | >20 |
| MMV1829350 | >20 | >20 | >20 | >20 |
| MMV691936 | >20 | >20 | >20 | >20 |
| MMV692115 | 10 | 10 | >20 | >20 |
| MMV691942 | >20 | >20 | >20 | >20 |
| MMV1593278 | 20 | 20 | >20 | >20 |
| MMV689463 | 20 | 20 | >20 | >20 |
| MMV1829361 | >20 | >20 | >20 | >20 |

**Table 1.** Continued.

| Compound ID | *Staphylococcus aureus* ATCC BAA-44 | *Staphylococcus aureus* clinical isolated | *Escherichia coli* ATCC BAA-198 | *Escherichia coli* clinical isolated |
|---|---|---|---|---|
| MMV1829412 | >20 | >20 | >20 | >20 |
| MMV693133 | >20 | >20 | >20 | >20 |
| MMV692623 | >20 | 20 | >20 | >20 |
| MMV692857 | 10 | 10 | 20 | >20 |
| MMV689635 | >20 | >20 | >20 | >20 |
| MMV689626 | >20 | >20 | >20 | >20 |
| MMV689625 | >20 | >20 | >20 | >20 |
| MMV1828776 | 2.5 | 2.5 | >20 | >20 |
| MMV1828058 | >20 | >20 | >20 | >20 |
| MMV1829114 | >20 | >20 | >20 | >20 |
| MMV1558323 | >20 | >20 | >20 | >20 |
| MMV1828860 | >20 | >20 | >20 | >20 |
| MMV1829111 | >20 | >20 | >20 | >20 |
| MMV674056 | >20 | >20 | >20 | >20 |
| MMV674593 | 20 | >20 | >20 | >20 |
| MMV893278 | >20 | >20 | >20 | >20 |
| MMV893256 | >20 | >20 | >20 | >20 |
| MMV1829290 | >20 | >20 | >20 | >20 |
| MMV1579936 | >20 | >20 | >20 | >20 |
| MMV1829556 | >20 | >20 | >20 | >20 |
| MMV1829523 | >20 | >20 | >20 | >20 |
| MMV1795510 | 20 | 20 | >20 | >20 |
| MMV1828026 | >20 | >20 | >20 | >20 |
| MMV1829658 | >20 | >20 | >20 | >20 |
| MMV1592761 | >20 | >20 | 20 | >20 |
| MMV674884 | >20 | >20 | >20 | >20 |
| MMV1848726 | >20 | >20 | >20 | >20 |

**Table 1.** Continued.

| Compound ID | *Staphylococcus aureus* ATCC BAA-44 | *Staphylococcus aureus* clinical isolated | *Escherichia coli* ATCC BAA-198 | *Escherichia coli* clinical isolated |
|---|---|---|---|---|
| MMV1829659 | >20 | >20 | >20 | >20 |
| MMV1829575 | >20 | >20 | >20 | >20 |
| MMV1829574 | >20 | >20 | >20 | >20 |
| MMV884814 | 20 | >20 | >20 | >20 |
| MMV693130 | 10 | 10 | >20 | >20 |
| MMV892517 | 20 | 10 | >20 | >20 |
| MMV1545678 | >20 | >20 | >20 | >20 |
| MMV1545674 | 20 | 20 | >20 | >20 |
| MMV1545675 | >20 | >20 | >20 | >20 |
| MMV1828832 | >20 | >20 | >20 | >20 |
| MMV1828843 | >20 | >20 | >20 | >20 |
| MMV1828705 | >20 | >20 | >20 | >20 |
| MMV1828056 | >20 | >20 | >20 | >20 |
| MMV1828766 | >20 | >20 | >20 | >20 |
| MMV1828728 | >20 | >20 | >20 | >20 |
| MMV1828816 | >20 | >20 | >20 | >20 |
| MMV1828810 | >20 | >20 | >20 | >20 |
| MMV1829345 | >20 | >20 | >20 | >20 |
| MMV893417 | 10 | 10 | >20 | >20 |
| MMV893415 | 20 | 20 | >20 | >20 |
| MMV897662 | 10 | 10 | >20 | >20 |
| MMV1579822 | >20 | >20 | >20 | >20 |
| MMV1828814 | >20 | >20 | >20 | >20 |
| MMV977436 | >20 | >20 | >20 | >20 |
| MMV1829460 | >20 | >20 | >20 | >20 |
| MMV892523 | 10 | 10 | 20 | >20 |
| MMV675568 | >20 | >20 | >20 | >20 |

**Table 1.** Continued.

| Compound ID | *Staphylococcus aureus* ATCC BAA-44 | *Staphylococcus aureus* clinical isolated | *Escherichia coli* ATCC BAA-198 | *Escherichia coli* clinical isolated |
|---|---|---|---|---|
| MMV1558324 | >20 | >20 | >20 | >20 |
| MMV688723 | >20 | >20 | >20 | >20 |

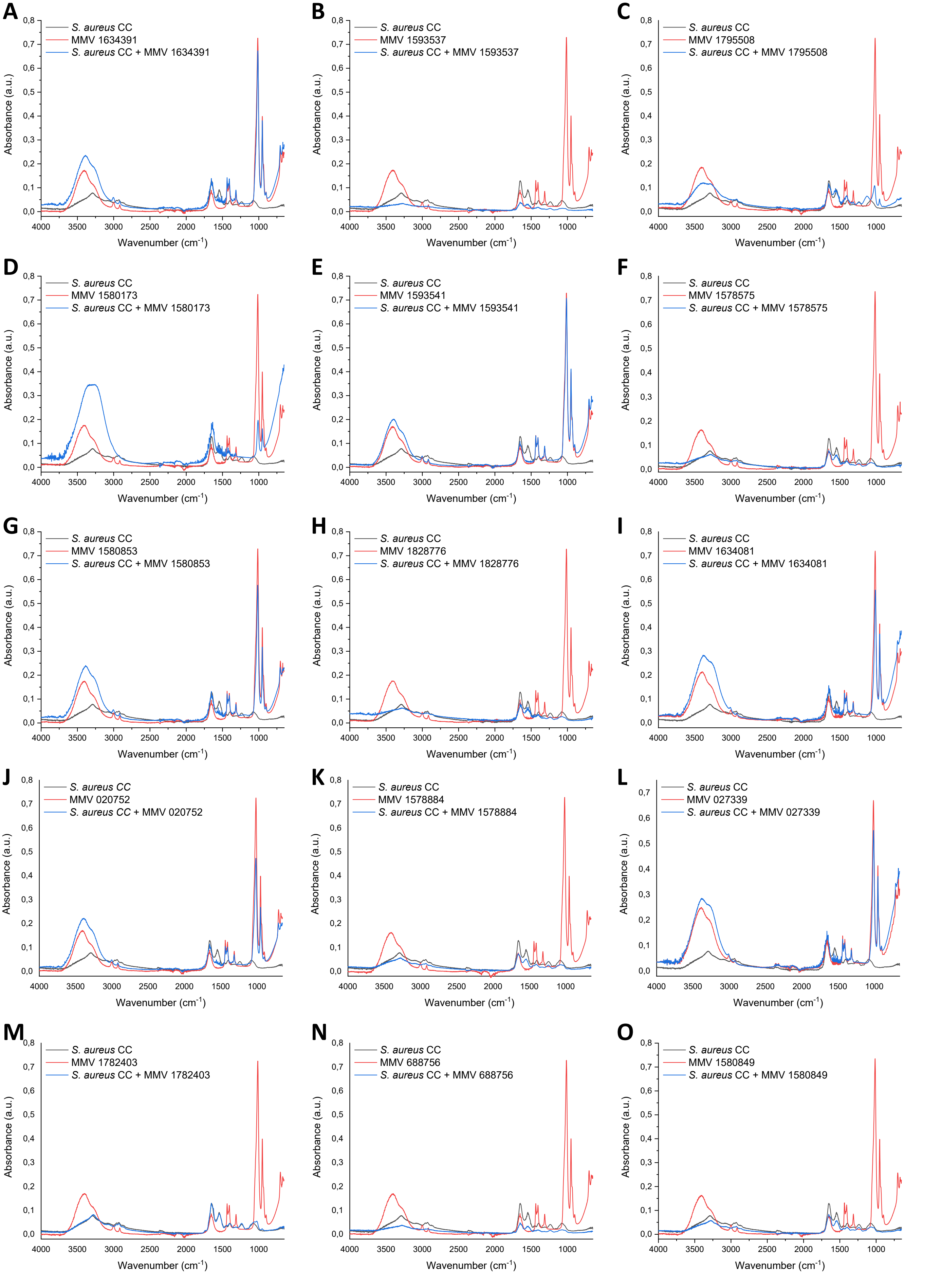


Figure S1: ATR-FTIR analysis reveals molecular shifts in the conformations of *S. aureus* CI alone and upon exposure to MMV compounds. Representative infrared spectra are displayed for bacterial samples (black line), MMV compounds (red line), and bacterial samples incubated with MMV compounds (blue line). (A-O) Full raw infrared spectrum for *S. aureus* CI, the respective MMV compound employed, and the bacterial-MMV complex.

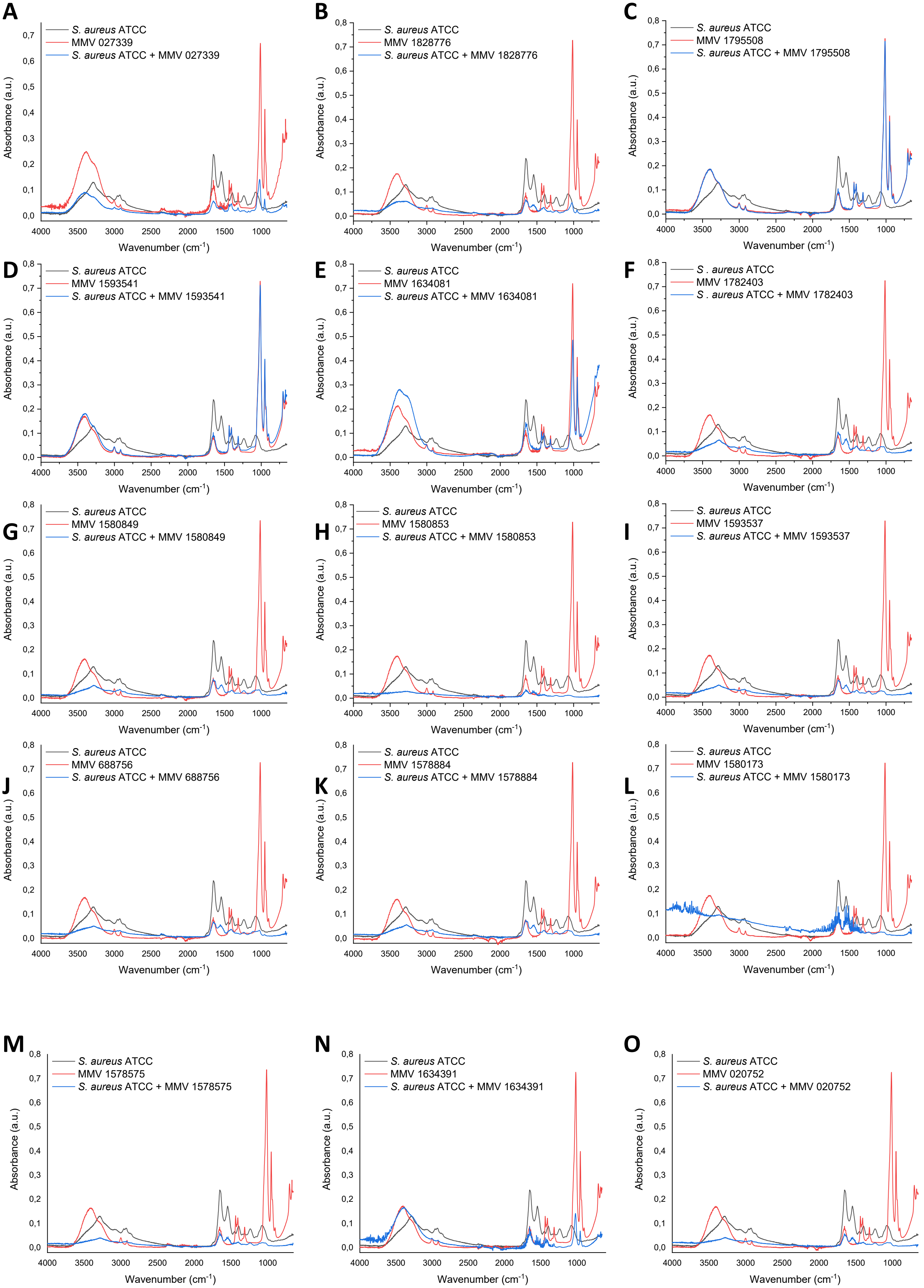


Figure S2: ATR-FTIR analysis reveals molecular shifts in the conformations of *S. aureus* ATCC alone and upon exposure to MMV compounds. Representative infrared spectra are displayed for bacterial samples (black line), MMV compounds (red line), and bacterial samples incubated with MMV compounds (blue line). (A-O) Full raw infrared spectrum for *S. aureus* ATCC*,* the respective MMV compound employed, and the bacterial-MMV complex.

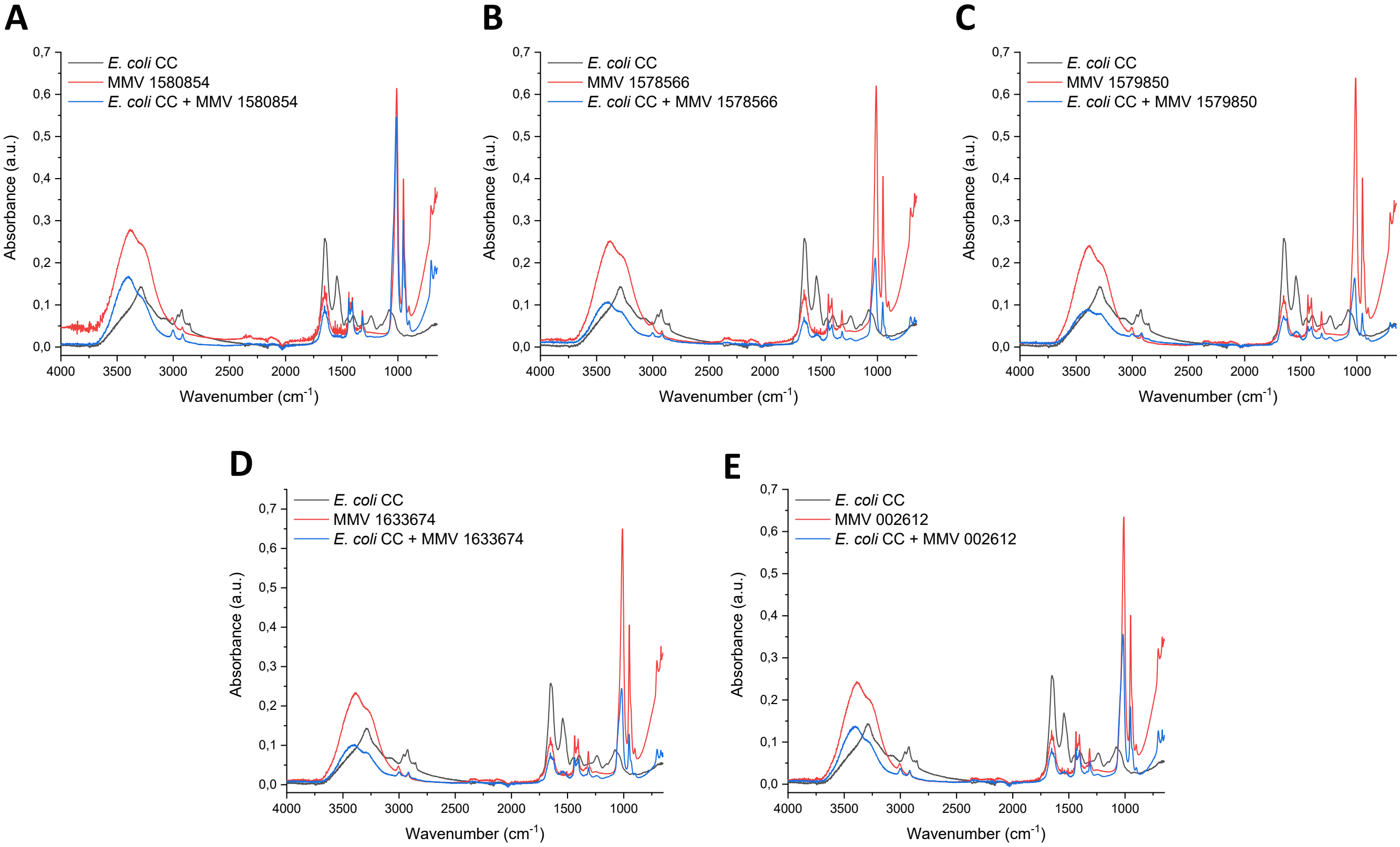


Figure S3: ATR-FTIR analysis reveals molecular shifts in the conformations of *E. coli* CI alone and upon exposure to MMV compounds. Representative infrared spectra are displayed for bacterial samples (black line), MMV compounds (red line), and bacterial samples incubated with MMV compounds (blue line). (A-E) Full raw infrared spectrum for *E. coli* CI, the respective MMV compound employed, and the bacterial-MMV complex.

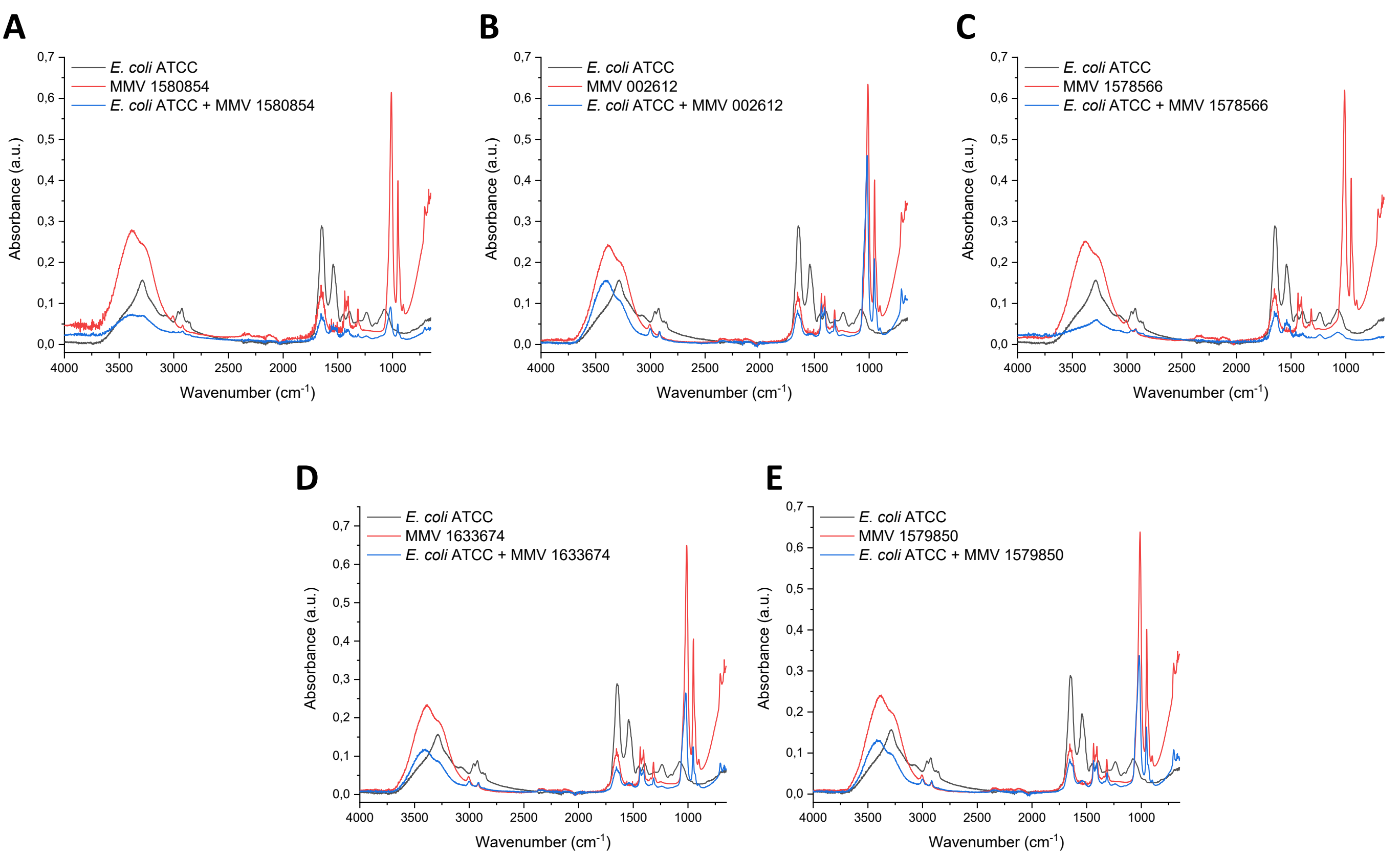


Figure S4: ATR-FTIR analysis reveals molecular shifts in the conformations of *E. coli* ATCC alone and upon exposure to MMV compounds. Representative infrared spectra are displayed for bacterial samples (black line), MMV compounds (red line), and bacterial samples incubated with MMV compounds (blue line). (A-E) Full raw infrared spectrum for *E. coli* ATCC*,* the respective MMV compound employed, and the bacterial-MMV complex.
